# Viral Manipulation of a Mechanoresponsive Signaling Axis Disassembles Processing Bodies

**DOI:** 10.1128/MCB.00399-21

**Published:** 2021-10-26

**Authors:** Elizabeth L. Castle, Carolyn-Ann Robinson, Pauline Douglas, Kristina D. Rinker, Jennifer A. Corcoran

**Affiliations:** a Department of Microbiology and Immunology, Dalhousie University, Halifax, Nova Scotia, Canada; b Department of Physiology and Pharmacology, University of Calgary, Calgary, Alberta, Canada; c Department of Chemical and Petroleum Engineering and Centre for Bioengineering Research and Education, University of Calgary, Calgary, Alberta, Canada; d Charbonneau Cancer Institute, University of Calgary, Calgary, Alberta, Canada; e Department of Microbiology, Immunology and Infectious Diseases, University of Calgary, Calgary, Alberta, Canada

**Keywords:** Kaposi's sarcoma-associated herpesvirus, RNA regulation, YAP, actin dynamics, herpesviruses, mechanotransduction, processing bodies, tumorigenesis, virus-host interactions

## Abstract

Processing bodies (PBs) are ribonucleoprotein granules important for cytokine mRNA decay that are targeted for disassembly by many viruses. Kaposi’s sarcoma-associated herpesvirus is the etiological agent of the inflammatory endothelial cancer, Kaposi’s sarcoma, and a PB-regulating virus. The virus encodes kaposin B (KapB), which induces actin stress fibers (SFs) and cell spindling as well as PB disassembly. We now show that KapB-mediated PB disassembly requires actin rearrangements, RhoA effectors, and the mechanoresponsive transcription activator, YAP. Moreover, ectopic expression of active YAP or exposure of ECs to mechanical forces caused PB disassembly in the absence of KapB. We propose that the viral protein KapB activates a mechanoresponsive signaling axis and links changes in cell shape and cytoskeletal structures to enhanced inflammatory molecule expression using PB disassembly. Our work implies that cytoskeletal changes in other pathologies may similarly impact the inflammatory environment.

## INTRODUCTION

Cells are exposed to a variety of environments, and they respond to changes in external force by adjusting internal tension. These mechanical cues can be transmitted to the cell through changes to extracellular contact nodes (focal adhesions) and contractile actomyosin structures to maintain tension homeostasis (1–5). Actin stress fibers (SFs) are cytoskeletal structures composed of thick actin bundles, often associated with focal adhesions (6), that are force responsive, maintaining cytoskeletal integrity in changing mechanical environments (7). SF formation is coordinated by the GTPase, RhoA; it activates the formin mammalian diaphanous protein 1 (mDia1) to promote actin filament growth and Rho-associated coiled-coil kinase (ROCK) to promote actomyosin contractility through nonmuscle myosin II (8–10). These RhoA effectors act together to promote the formation of contractile and stable actin filaments in response to mechanical and chemical stimuli (11).

External forces elicit a cascade of signals using actin as force transducers to alter gene expression. Activated serum response factor (SRF) transcription responds to actin polymerization (12). SRF activation is negatively regulated by the cytoplasmic concentration of monomeric globular actin (G-actin) (13). However, inducers of filamentous actin (e.g., active RhoA) deplete G-actin levels, leading to SRF nuclear translocation and transcription (13). A more recent example is the mechanoresponsive transcriptional coactivator Yes-associated protein (YAP), whose activity can be controlled by cell shape and cytoskeletal structure (14–17). YAP is nuclear and active in response to low cell-cell contact (18), high stiffness of the extracellular matrix (ECM) (14) in shear stress due to fluid flow (19–23), or after G protein-coupled receptor (GPCR) activation (17). Most of these signals induce the activity of RhoA and promote the formation of SFs (24, 25), implicating actin cytoskeletal structures as requisite intermediates for YAP activation.

Nuclear YAP associates with its coactivators to mediate transcription of genes involved in cell proliferation, differentiation, survival, and migration (16). Consistent with this, nuclear YAP is often protumorigenic and drives progression of many oncogenic traits in a variety of cancers. These include the induction of cell stemness (26), altered metabolism (27), cancer cell invasion/vascular remodeling (28–30), and altered growth and proliferation (31–33). Kaposi’s sarcoma (KS) is an endothelial cell (EC) cancer that is strongly linked to Kaposi’s sarcoma-associated herpesvirus (KSHV) (34–37). KSHV establishes persistent, lifelong infection of its human host and displays two types of infection, latent and lytic. In KS, the majority of the tumor ECs are latently infected, while lytic replication is rare, in part because these cells die as a result of viral replication (38–41). That said, during their short lifetime, lytic cells expel progeny virus and secrete large quantities of proinflammatory and angiogenic molecules, making even infrequent lytic replication an important driver of KS. A key contributor to this secretory phenotype is the constitutively active viral G protein-coupled receptor (vGPCR), a lytic viral protein (42, 43). Despite the paracrine contributors like vGPCR, the few gene products that are expressed during the KSHV latent cycle are central for viral tumorigenesis. Many features of *in vivo* KS are recapitulated by *in vitro* latent infection of primary ECs or ectopic expression of individual KSHV latent genes, including enhanced proliferation and an elongated or “spindled” morphology characteristic of KS. Spindling is induced by two KSHV latent genes, vFLIP (44) and kaposin B (KapB) (45). Spindled cells also secrete a variety of proinflammatory cytokines and angiogenic factors to further promote tumor progression through inflammatory cytokine production (44, 46–49). However, no information exists to demonstrate YAP involvement in KSHV latency, despite the fact that the vGPCR has been shown to activate YAP during KSHV lytic infection (50).

One way that KSHV latency promotes the proinflammatory and protumorigenic KS microenvironment is via KapB-mediated disassembly of cytoplasmic ribonucleoprotein granules called processing bodies (PBs) (45). PBs are involved in many RNA regulatory processes such as RNA silencing, nonsense-mediated decay, and mRNA decay and translational suppression of mRNA (51–55). We and others have shown that PBs are the major site for the translational suppression or constitutive decay of human mRNAs that code for potent regulatory molecules, such as proinflammatory cytokines (45, 56–58). There are ∼4,500 of these transcripts, all of which bear destabilizing AU-rich elements (AREs) in their 3′ untranslated regions (3′ UTRs) (56, 59–65). PB abundance and composition are extremely dynamic and respond to cellular stress (66–69). Specifically, activation of the stress-responsive p38/MK2 MAP kinase pathway by KapB elicits PB disassembly and prevents constitutive ARE-mRNA turnover or translation suppression (43, 45, 62, 70, 71). This is an important yet underappreciated regulatory mechanism that fine-tunes the production of potent proinflammatory cytokines and angiogenic factors in KS.

Though PBs are dynamic and stress responsive, the precise signaling events that lead to PB assembly or disassembly are not well understood. We showed previously that KapB binds and activates MK2, which then phosphorylates hsp27, complexes with p115RhoGEF, and activates RhoA to elicit PB disassembly (45, 72, 73). While it is well established that RhoA coordinates SF formation (11, 74–76), the precise mechanism of how RhoA promotes PB disassembly is not appreciated (45, 69). In an effort to better understand the regulation of PB disassembly by KapB and RhoA, we began by targeting downstream RhoA effectors reported to promote SF formation to determine if the proteins known to mediate cytoskeletal remodeling were also necessary for PB disassembly. We reasoned that, at some point, we would be able to uncouple the signaling events that led to SFs from those that led to PB disassembly. We were not. We now present data that conclusively shows KapB-mediated PB disassembly is dependent not only on RhoA but on cytoskeletal structures, actomyosin contractility, and the presence of the mechanoresponsive transcription transactivator, YAP. We also present the first evidence of the involvement of YAP in the tumorigenic phenotypes induced by a KSHV latent gene, KapB. We also extend these studies beyond their impact on viral tumorigenesis by determining the mechanical regulation of PB dynamics in the absence of KapB expression, and we show that induced cell contractility, cytoskeletal structures, and active YAP all precede PB disassembly. Using a viral protein from an oncogenic virus, we have discovered a mechanoresponsive signaling pathway that transduces signals from cell shape and cytoskeletal structures to YAP to control PBs, posttranscriptional regulators of cellular gene expression.

## RESULTS

### RhoA effectors controlling SF formation are required for PB disassembly.

**FIG 1:**
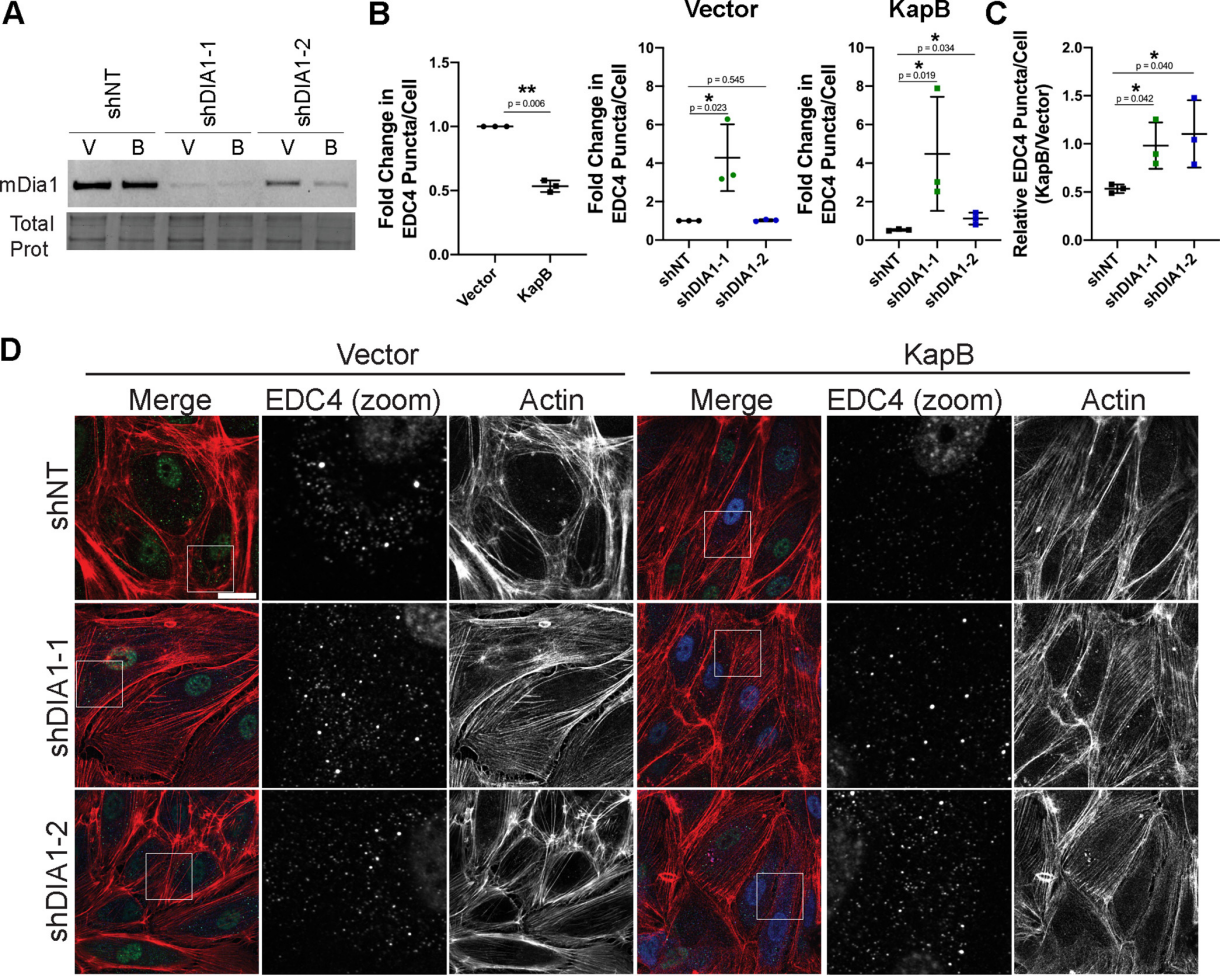
The RhoA-effector mDia1 is required for KapB-mediated PB disassembly. KapB- and vector-expressing HUVECs were transduced with shRNAs targeting mDia1 (shDIA1-1 and shDIA1-2) or with a nontargeting (shNT) control and selected. In parallel, cells were fixed for immunofluorescence or lysed for immunoblotting. (A) One representative immunoblot of three independent experiments stained with mDia1-specific antibody is shown. (B and C) Fixed cells were stained for CellProfiler analysis as detailed in Materials and Methods. (B) The number of EDC4 puncta per cell was quantified and normalized to the vector nontargeting (NT) control within each replicate. (C) CellProfiler data were used to calculate the ratio of EDC4 puncta counts in KapB-expressing cells versus the vector control for each treatment condition. (D) Representative images of cells stained for PB resident proteins EDC4 (green), KapB (blue), and F-actin (red, phalloidin). Boxes indicate area shown in the EDC4 (zoom) panel. Scale bar represents 20 μm. Statistics were determined using ratio-paired *t* tests between control and experimental groups; error bars represent standard deviation. *n* = 3 independent biological replicates. *, *P* < 0.05; **, *P* < 0.01.

We previously showed that KapB-mediated PB disassembly required RhoA (45). In this work, we investigated whether downstream RhoA effectors known to control SF formation also control PB disassembly. Mammalian diaphanous protein 1 (mDia1) and Rho-associated coiled-coil kinase (ROCK) are considered the main coordinators of RhoA-mediated SF formation (11, 77). mDia1 is a formin that promotes actin filament polymerization (11). To examine whether mDia1 was required for KapB-mediated PB disassembly, we designed short hairpin RNAs (shRNAs) to silence mDia1 mRNA. KapB- and vector-expressing human umbilical vein endothelial cells (HUVECs) were transduced with mDia1-targeting shRNAs and selected. Silencing efficacy was confirmed with immunoblotting (Fig. 1A). PB analysis was performed using CellProfiler to quantify immunofluorescence images stained for the hallmark PB resident protein, enhancer of mRNA decapping 4 (EDC4), as described in detail in Materials and Methods (78, 79). Knockdown of mDia1 increased PBs in KapB-expressing cells (Fig. 1B and D). mDia1-sh1 showed a greater increase in PBs than mDia1-sh2 (Fig. 1B) and also increased PBs in vector control cells, likely because mDia1-sh1 reduced protein expression by 90%, whereas mDia1-sh2 reduced it by 40 to 50% (Fig. 1A). To separate how silencing of mDia 1 impacted baseline turnover of PBs from how silencing impacts KapB-mediated PB disassembly, we calculated the ratio of PBs per cell in KapB-expressing cells to PBs per cell in the control. This is important because this calculation shows whether KapB is still able to disassemble PBs, relative to vector, in the context of mDia 1 silencing. If the ratio is ≥1 after sh-mDia 1 treatment, it indicates that KapB is no longer able to disassemble PBs in comparison to the vector control and that mDia 1 contributes directly to KapB-mediated PB disassembly. Conversely, if the ratio is ∼0.4 to 0.6, it indicates that KapB can still disassemble PBs, even in the context of sh-mDia 1 treatment. In this case, we determined that silencing using both mDia1-sh1 and mDia1-sh2 restored the PB ratio in KapB/vector cells to ∼1, indicating that the ability of KapB to disassemble PBs is lost after mDia 1 silencing and that this is a specific effect (Fig. 1C). We note that this ratio will be reported in subsequent figures for every RNA silencing or drug treatment applied to test KapB-mediated PB disassembly. We also observed that mDia1 silencing did not eliminate SF formation (Fig. 1D) but, instead, increased elongated cells with visible actin SFs across the cell in both vector and KapB conditions. The visible actin structures may represent different SF subtypes or actin bundles that compensate for the loss of mDia1 (76).

**FIG 2:**
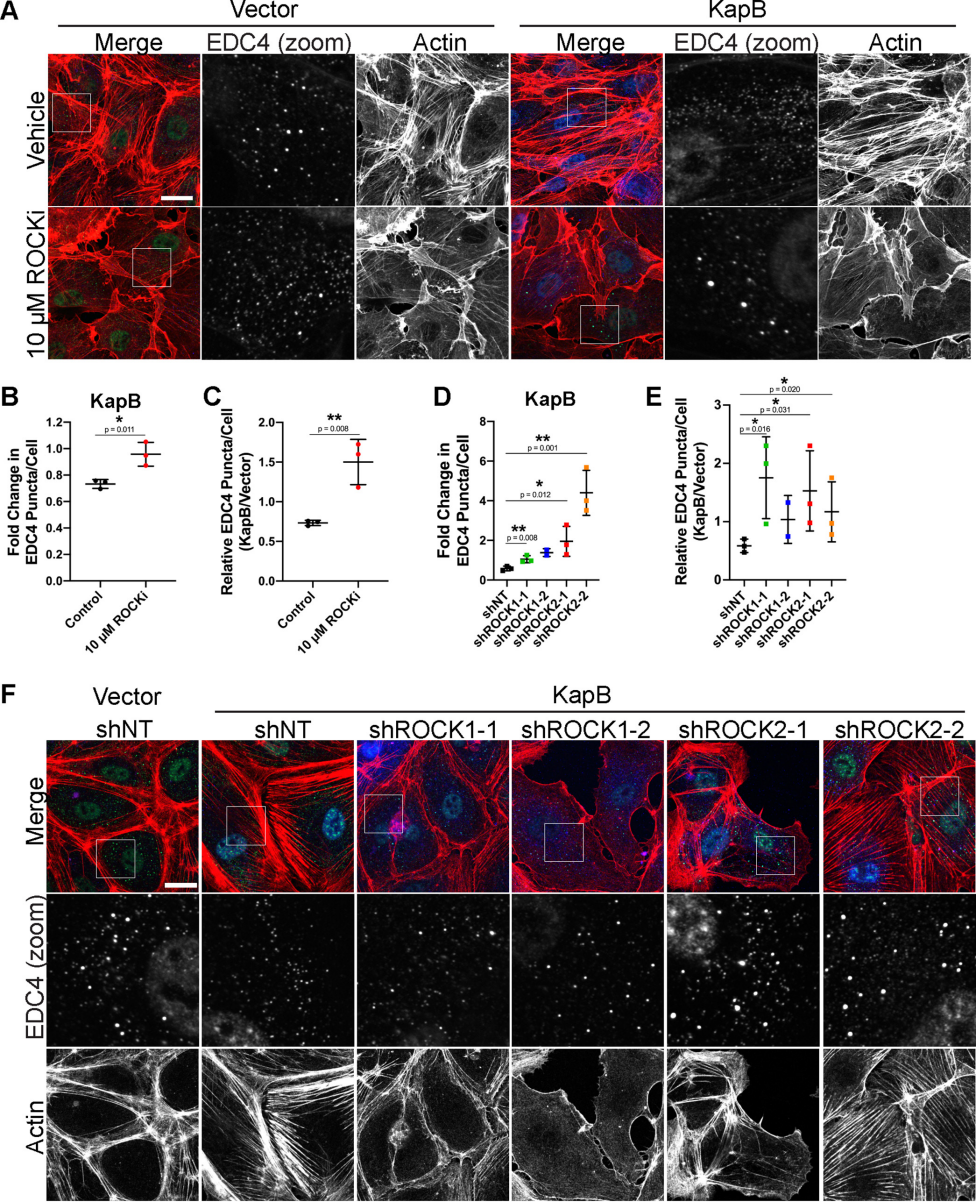
The RhoA effector ROCK is required for KapB-mediated PB disassembly. (A to C) KapB- and vector-expressing HUVECs were treated with 10 μM Y-27632 or water control for 4 h and fixed for immunofluorescence. (A) Representative images of cells stained for PB resident proteins EDC4 (green), KapB (blue), and F-actin (red, phalloidin). Boxes indicate area shown in the EDC4 (zoom) panel. Scale bar represents 20 μm. (B and C) Fixed cells were stained for CellProfiler analysis as detailed in Materials and Methods. (B) The number of EDC4 puncta per cell was quantified and normalized to the vector NT control within each replicate. (C) CellProfiler data were used to calculate the ratio of EDC4 puncta counts in KapB-expressing cells versus the vector control for each treatment condition. (D to F) KapB- and vector-expressing HUVECs were transduced with shRNAs targeting ROCK1 and ROCK2 (shROCK1-1, shROCK1-2, shROCK2-1, and shROCK2-2) or with a nontargeting (shNT) control and selected. Cells were fixed for immunofluorescence. (D and E) Fixed cells were stained for CellProfiler analysis as detailed in Materials and Methods. (D) The number of EDC4 puncta per cell was quantified and normalized to the vector NT control within each replicate. (E) CellProfiler data were used to calculate the ratio of EDC4 puncta counts in KapB-expressing cells versus the vector control for each treatment condition. (F) Representative images of cells stained for PB resident proteins EDC4 (green), KapB (blue), and F-actin (red, phalloidin). Boxes indicate images shown in EDC4 (zoom) panel. Scale bar represents 20 μm. Statistics were determined using ratio-paired *t* tests between control and experimental groups; error bars represent standard deviation from *n* = 3 independent biological replicates except shROCK1-2 (*n* = 2). *, *P* < 0.05; **, *P* < 0.01.

**FIG 3:**
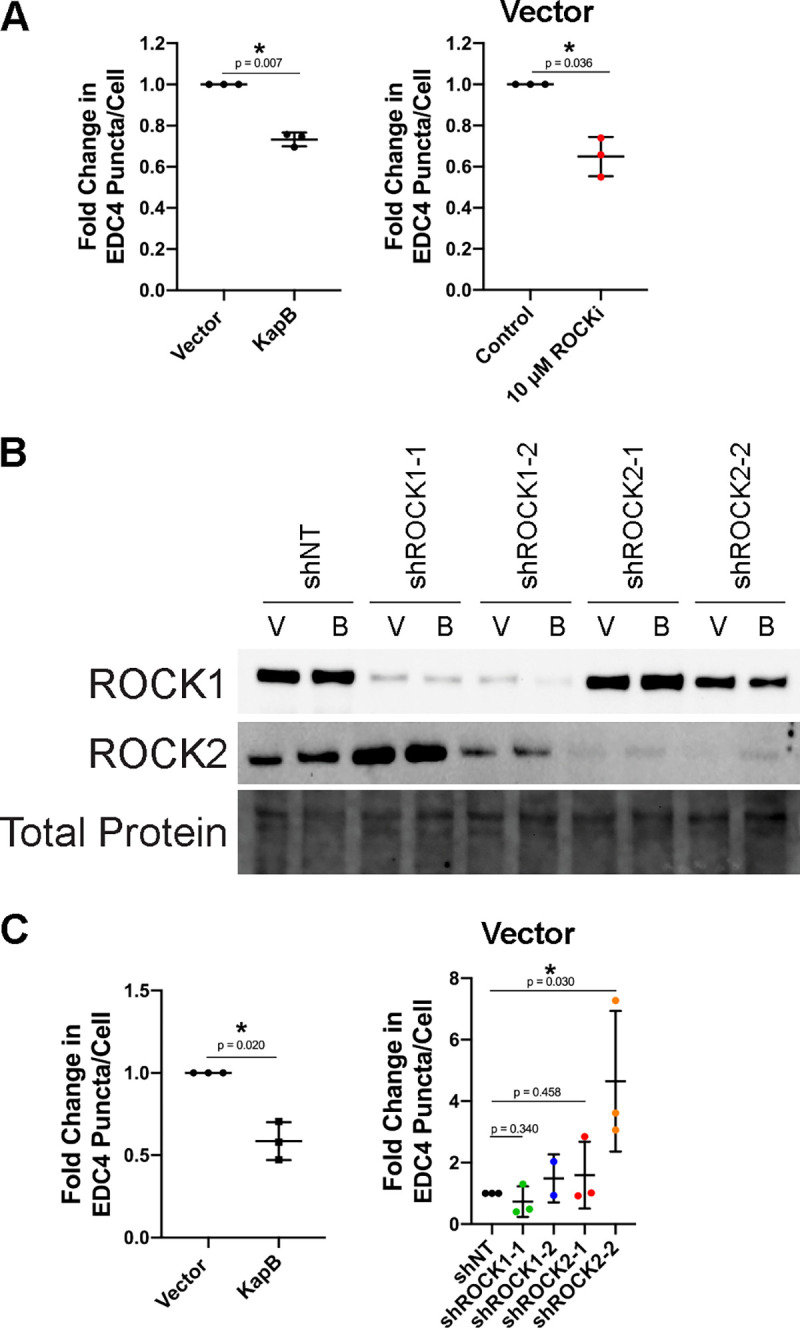
The RhoA-effector ROCK is required for KapB-mediated PB disassembly, knockdown confirmation, and vector data. (A) KapB- and vector-expressing HUVECs were treated with 10 μM Y-27632 or water control for 4 h and fixed for immunofluorescence. Fixed cells were stained for CellProfiler analysis as detailed in Materials and Methods. The number of EDC4 puncta per cell was quantified and normalized to the vector control. Vector control data are shown. (B and C) KapB- and vector-expressing HUVECs were transduced with shRNAs targeting ROCK1 and ROCK2 (shROCK1-1, shROCK1-2, shROCK2-1, and shROCK2-2) or with a nontargeting (shNT) control and selected. In parallel, cells were lysed for immunoblotting or fixed for immunofluorescence. (B) One representative immunoblot of three independent experiments stained using ROCK1- and ROCK2-specific antibodies. (C) Fixed cells were stained for CellProfiler analysis as detailed in Materials and Methods. The number of EDC4 puncta per cell was quantified and normalized to the vector NT control within each replicate. Vector control data are shown. Statistics were determined using a ratio-paired *t* test between control and experimental groups; error bars represent standard deviation. *n* = 3 independent biological replicates. *, *P* < 0.05.

ROCK promotes SF formation by increasing actin contractility and inhibiting actin-severing activity (80). Chemical inhibition of both isoforms of ROCK, ROCK1 and ROCK2, with Y-27632 (81) restored PBs in KapB-expressing cells and increased the ratio of KapB/vector PBs (Fig. 2A to C). HUVECs treated with Y-27632 had scalloped cell edges with minimal actin structure (Fig. 2A). Y-27632 did not visibly alter cell viability during the indicated treatment. To determine whether PB disassembly is dependent on a single ROCK isoform, both ROCK1 and ROCK2 were knocked down with isoform-specific shRNAs. Knockdown efficacy was confirmed with immunoblotting (Fig. 3). Independent knockdown of ROCK1 and 2 increased PB counts in KapB-expressing cells (Fig. 2D and F) and restored the ratio of KapB to vector PB counts (Fig. 2E). This indicated that both ROCK1 and ROCK2 can contribute to KapB-mediated PB disassembly. ROCK2 knockdown showed more robust PB restoration, both in terms of PB counts and PB size, than that seen with ROCK1 knockdown (Fig. 2D and F). Quantification of PB counts in control cells for both pan-ROCK inhibition and ROCK knockdown experiments is reported in Fig. 3. While pan-ROCK inhibition and ROCK1 knockdown treatments both eliminate SFs, ROCK2 knockdown retains pronounced actin fibers in the cells (Fig. 2F). Similar to mDia1 knockdown, this may indicate a compensatory mechanism to retain cell shape and suggests that only a subset of SFs may be required for PB disassembly. Taken together, these data show that inhibition of RhoA effectors that mediate SF formation can reverse KapB-mediated PB disassembly. Put another way, we have been unable to uncouple KapB-mediated SF formation from KapB-mediated PB disassembly.

**FIG 4:**
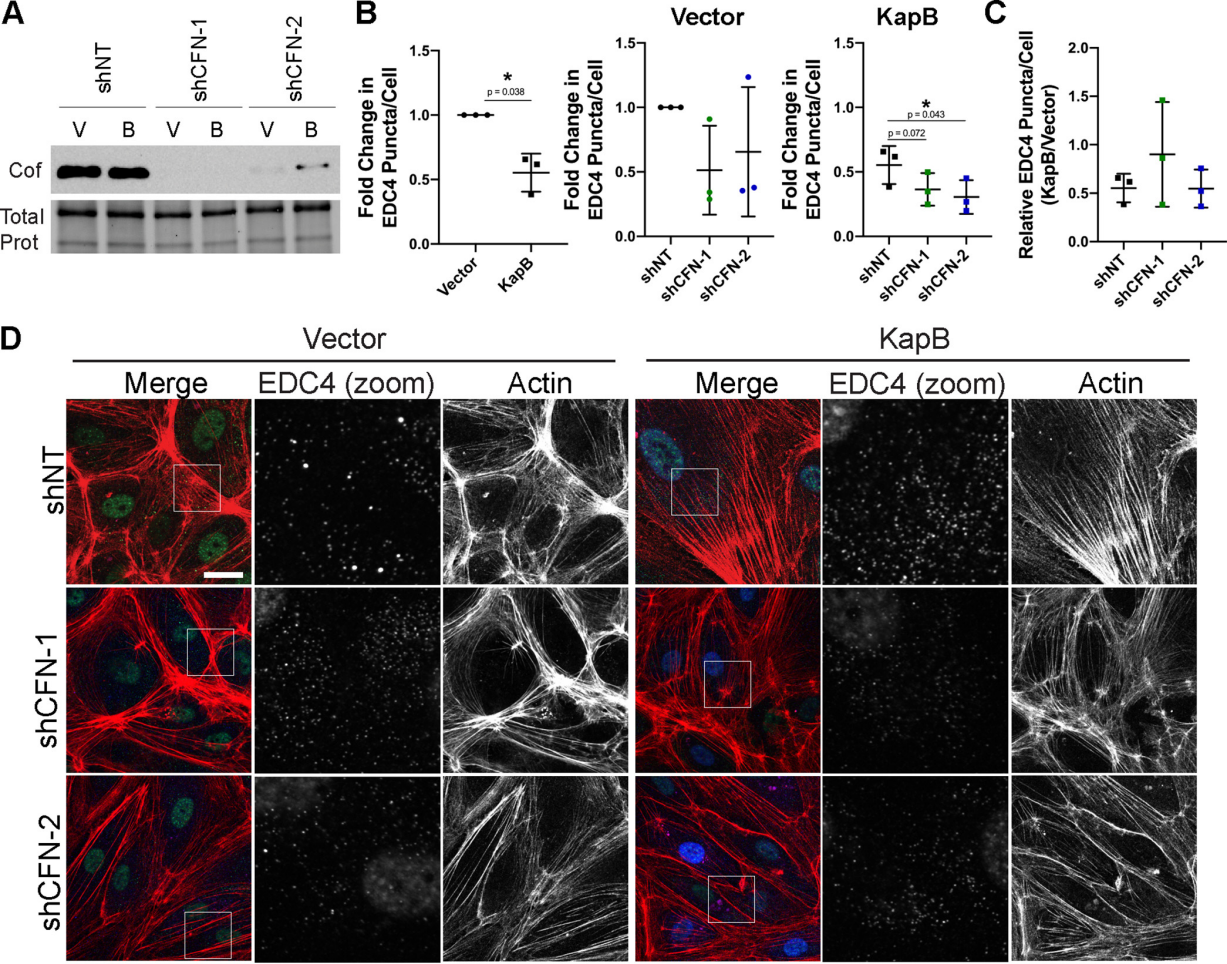
Cofilin knockdown augments KapB-mediated PB disassembly. KapB- and vector-expressing HUVECs were transduced with shRNAs targeting cofilin (shCFN-1 and shCFN-2) or with a nontargeting (shNT) control and selected. In parallel, cells were fixed for immunofluorescence or lysed for immunoblotting. (A) One representative immunoblot of three independent experiments stained using a cofilin-specific antibody. (B and C) Fixed cells were stained for CellProfiler analysis as detailed in Materials and Methods. (B) The number of EDC4 puncta per cell was quantified and normalized to the vector NT control within each replicate. (C) CellProfiler data were used to calculate the ratio of EDC4 puncta counts in KapB-expressing cells versus the vector control for each treatment condition. (D) Representative images of cells stained for PB resident proteins EDC4 (green), KapB (blue), and F-actin (red, phalloidin). Boxes indicate the area of the field of view that is shown in EDC4 (zoom) panel. Scale bar represents 20 μm. Statistics were determined using a ratio-paired *t* test between control and experimental groups; error bars represent standard deviation. *n* = 3 independent biological replicates. *, *P* < 0.05.

ROCK phosphorylates and activates LimK, which then phosphorylates and inactivates cofilin, an actin-severing protein (82). In this way, ROCK promotes SF formation by inactivating cofilin. To investigate the role of cofilin in KapB-mediated PB disassembly, shRNAs to knockdown cofilin expression were used (Fig. 4A). Since ROCK activation results in less cofilin activity and reduced actin severing, we hypothesized that knockdown of cofilin in KapB-expressing cells would augment KapB-mediated PB disassembly. Knockdown of cofilin resulted in elongated cells with more SFs in both control and KapB-expressing cells (Fig. 4D). Cofilin knockdown also induced PB disassembly in control cells and aided PB disassembly in KapB-expressing cells (Fig. 4B and C). This indicates that inhibition of cofilin elicits PB disassembly and supports the hypothesis that reducing cofilin activity is one of the reasons that KapB promotes SF formation and PB disassembly.

### G-actin concentration does not influence PB disassembly.

**FIG 5:**
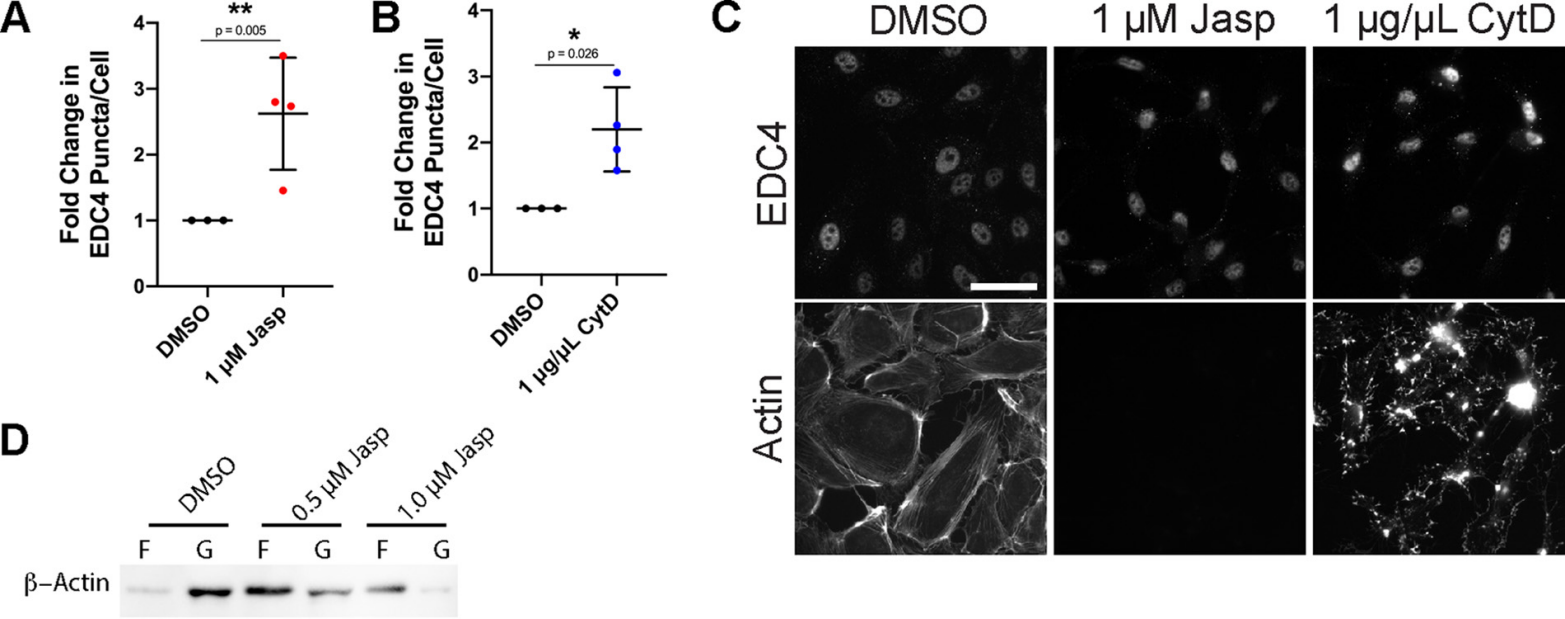
G-actin concentration does not control PB disassembly. (A to C) HUVECs were treated with 1 μM Jasp (polymerizes actin and decreases monomeric G-actin), 1 μg/μl CytD (actin depolymerization to increase monomeric G-actin), or a DMSO control for 30 min. (A and B) Fixed cells were stained for CellProfiler analysis as detailed in Materials and Methods. The number of EDC4 puncta per cell was quantified and normalized to the DMSO control. (C) Representative images of cells stained for PB resident proteins EDC4 and F-actin (phalloidin). Actin is not seen in Jasp panel due to Jasp-mediated interference with phalloidin staining (86). Scale bar represents 20 μm. Statistics were determined using a ratio-paired *t* test between control and experimental groups; error bars represent standard deviation. *n* = 3 independent biological replicates. (D) Representative immunoblot of filamentous and globular actin fractions separated by ultracentrifugation as detailed in Materials and Methods from HUVECs treated with DMSO, 0.5 or 1.0 μM Jasp. *, *P* < 0.05; **, *P* < 0.01.

Since we could not uncouple the signaling controlling SF formation from PB disassembly, we investigated whether changes in the concentration of monomeric G-actin, known to control cellular stress and SRF transcriptional responses (13, 83), could be controlling PBs. Several studies have shown that increasing the proportion of filamentous actin decreases the cytoplasmic concentration of monomeric G-actin (83–85). We investigated if our phenotype, PB disassembly, was controlled by changes in the proportion of monomeric G-actin. To determine this, cells were treated with drugs known to either decrease or increase the proportion of monomeric G-actin. Jasplakinolide (Jasp) treatment decreases the G-actin fraction by facilitating actin nucleation and aberrant polymerization of actin (86). Conversely, the actin polymerization inhibitor cytochalasin D (CytD) caps the barbed end of actin filaments, preventing further elongation of the actin filament and increasing the free G-actin concentration (87). Jasp treatment, which prevents imaging of actin structure due to interference with phalloidin (Fig. 5C) (86), resulted in an increased F-actin fraction in HUVECs (Fig. 5B). CytD treatment resulted in HUVECs with little to no filamentous actin structure (Fig. 5C). If the level of G-actin is the signal that regulates PB dynamics, we hypothesized that Jasp, which decreases G-actin levels, would mediate PB disassembly, while CytD would do the opposite and promote PB assembly. However, both treatments increased the PB count per cell (Fig. 5A to C); these data indicate that the concentration of G-actin does not influence PB disassembly, and this is not the mechanism by which actin SF formation or enhanced activity of RhoA alters PB dynamics. These data are congruent with our mDia1 and ROCK knockdown experiments that show retention of visible F-actin bundles despite PB restoration.

### α-Actinin-1 activity promotes PB disassembly.

**FIG 6:**
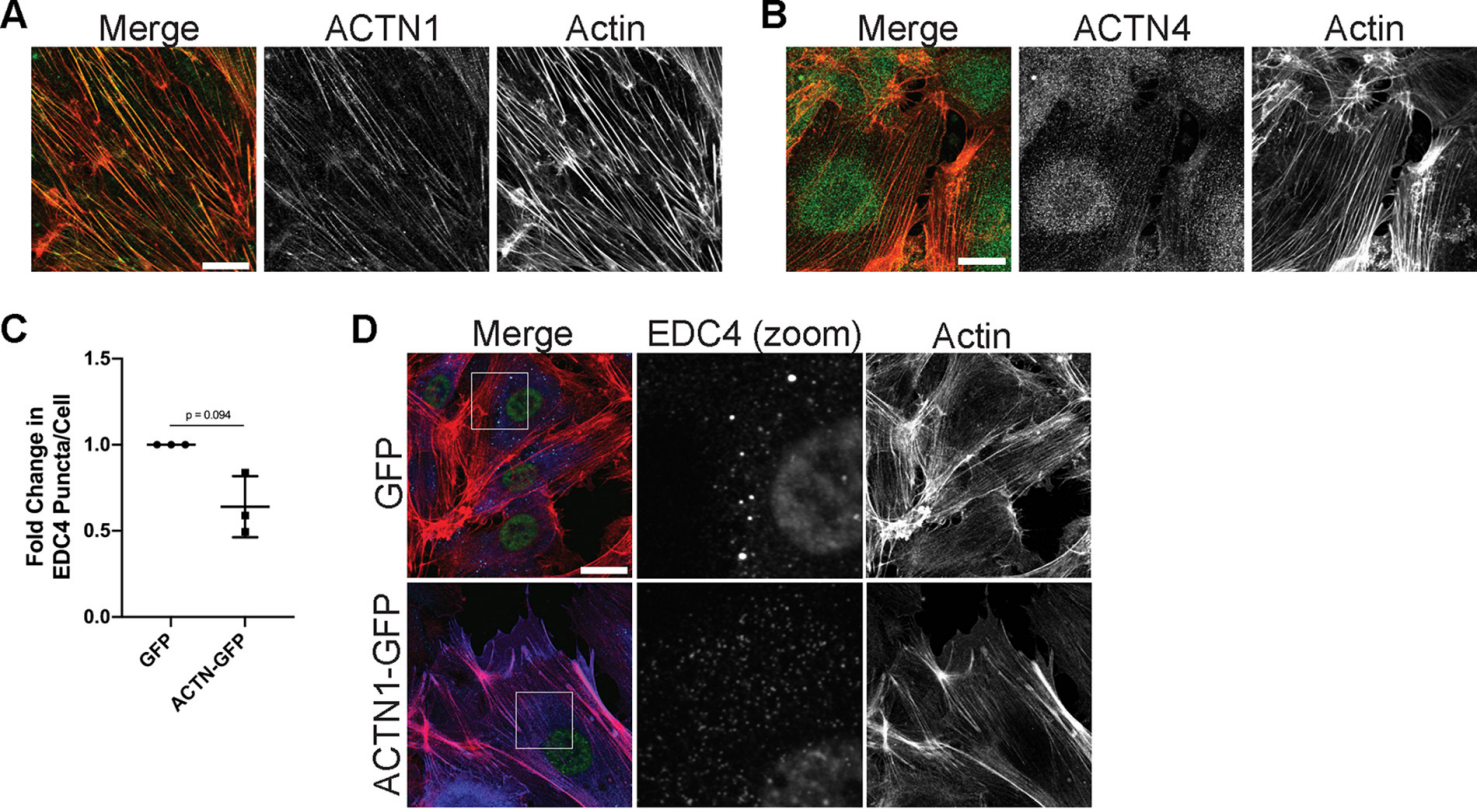
α-Actinin-1 overexpression mediated SF formation and PB disassembly. HUVECs were fixed and stained with antibodies for α-actinin-1 (A) and α-actinin-4 (B). (C and D) HUVECs transduced with recombinant lentiviruses expressing GFP-tagged α-actinin-1 (ACTN-GFP) or a GFP control were selected and fixed for immunofluorescence. (C) Fixed cells were stained for CellProfiler analysis as detailed in Materials and Methods. The number of EDC4 puncta per cell was quantified and normalized to the vector GFP control. (D) Representative images of cells stained for PB resident proteins EDC4 (false-colored green), ACTN-GFP (false-colored blue), and F-actin (red, phalloidin). Boxes indicate images shown in EDC4 (zoom) panel. Scale bar represents 20 μm. Statistics were determined using a ratio-paired *t* test between control and experimental groups; error bars represent standard deviation. *n* = 3 independent biological replicates. *, *P* < 0.05.

The actinins are primarily known for their role in bundling actin fibers, though in nonmuscle cells, α-actinin-1 and 4 do not mediate actin bundling to the same extent (88). α-Actinin-4 can, at times, localize to dorsal SFs, but it primarily mediates focal adhesion turnover and can act as a transcriptional regulator of genes associated with cell proliferation and differentiation (89–91). α-Actinin-1 primarily mediates SF bundling and formation, as well as focal adhesion maturation (89, 90). Using immunofluorescence, we observed that the localization of the two isoforms seen in HUVECs (Fig. 6A and B) was consistent with the reported localization and function, as α-actinin-1 was localized to actin fibers, and α-actinin-4 was more diffusely cytoplasmic and nuclear, with some actin fiber localization (89, 90). Since α-actinin-1 associated with SFs in HUVECs and overexpression of α-actinin–green fluorescent protein (GFP) has been shown to localize and reinforce SFs (92, 93), we asked whether its overexpression would promote PB disassembly. Overexpression of ACTN1-GFP resulted in elongated ECs with large SFs (Fig. 6C). ACTN1-GFP decreased EDC4 puncta per cell, suggesting that enhancing SF bundling and focal adhesion maturation positively regulates PB disassembly (Fig. 6C and D).

### Changes in cytoskeletal contractility control PB disassembly.

**FIG 7:**
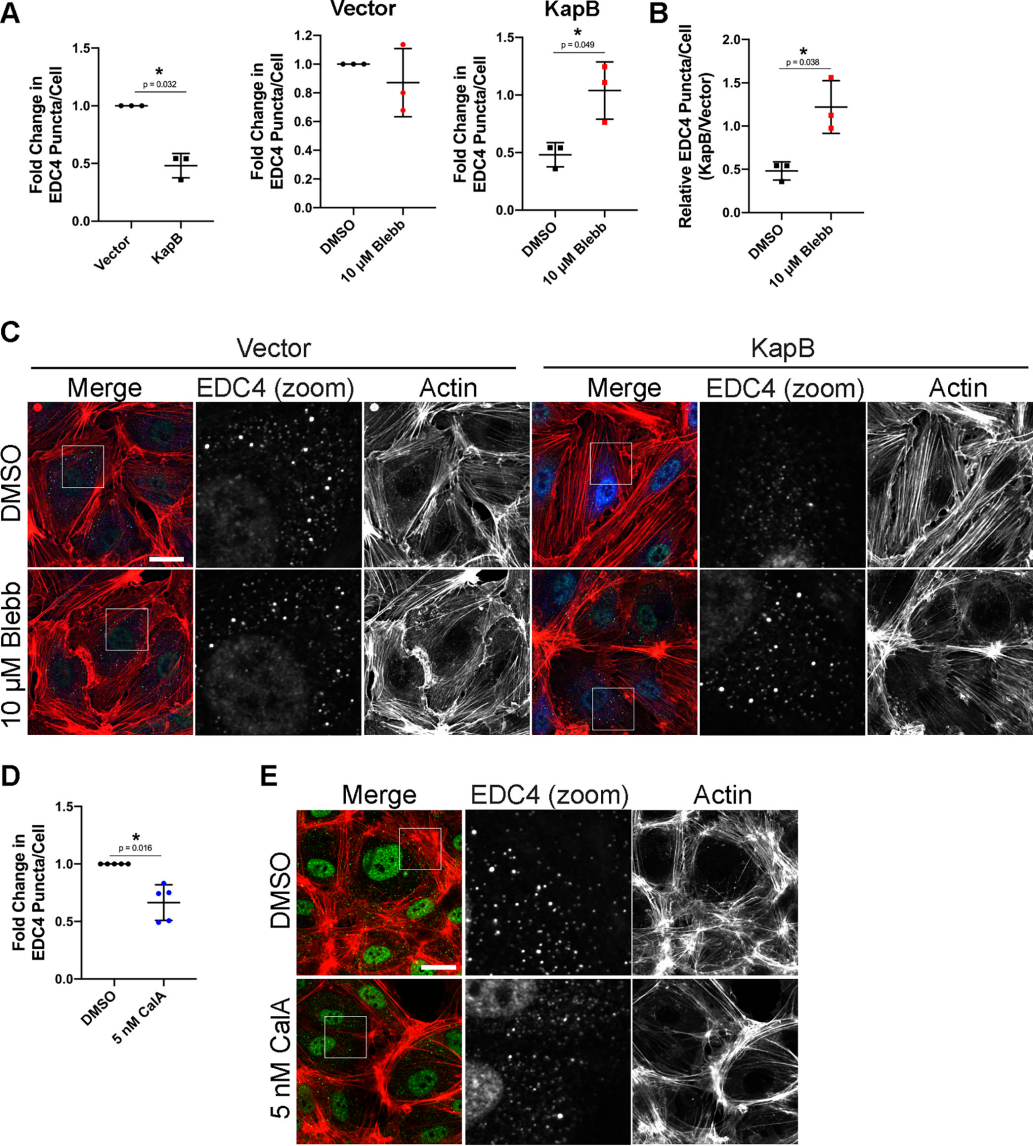
Actomyosin contractility controls PB disassembly. (A to C) KapB- and vector-expressing HUVECs were treated with 10 μm blebbistatin to inhibit actomyosin contractility or DMSO for 30 min and fixed for immunofluorescence. (A and B) Fixed cells were stained for CellProfiler analysis as detailed in Materials and Methods. (A) The number of EDC4 puncta per cell was quantified and normalized to the vector NT control within each replicate. (B) CellProfiler data were used to calculate the ratio of EDC4 puncta counts in KapB-expressing cells versus the vector control for each treatment condition. (C) Representative images of cells stained for PB resident proteins EDC4 (green), KapB (blue), and F-actin (red, phalloidin). Boxes indicate area shown in the EDC4 (zoom) panel. Scale bar represents 20 μm. (D and E) Untransduced HUVECs were treated with 5 nM calyculin A (CalA) to stimulate actomyosin contraction or DMSO for 20 min and fixed for immunofluorescence. (D) Fixed cells were stained for CellProfiler analysis as detailed in Materials and Methods. EDC4 puncta per cell were quantified and normalized to the DMSO control within each replicate. (E) Representative images of cells treated with 5 nM CalA and stained for PB resident proteins EDC4 (green) and F-actin (red, phalloidin). Boxes indicate area shown in the EDC4 (zoom) panel. Scale bar represents 20 μm. Statistics were determined using ratio-paired *t* tests between control and experimental groups; error bars represent standard deviation. *n* = 3 (A and B) and *n* = 5 (D) independent biological replicates. *, *P* < 0.05.

One of the downstream activities of the kinase, ROCK, is to phosphorylate myosin light chain to induce nonmuscle myosin II (NMII)-mediated actomyosin contraction (94). Since ROCK is required for KapB-mediated PB disassembly, we determined whether functional actomyosin contractility is also required. KapB-expressing cells were treated with blebbistatin, which inhibits NMII-mediated actomyosin contractility by maintaining NMII in a conformation that is unable to bind actin filaments (95). Treatment of KapB-expressing cells with blebbistatin restored both PB levels in KapB-expressing cells, as well as the KapB/vector ratio of PBs (Fig. 7A to C), indicating that contractility is required for KapB-induced PB disassembly. ECs treated with blebbistatin had normal actin structure (Fig. 7C). Blebbistatin treatment for the indicated time did not visibly alter cell viability. To determine if contraction would elicit the same phenotype in the absence of KapB, cells were treated with calyculin A (CalA), an inhibitor of myosin light chain phosphatase that promotes NMII phosphorylation and actomyosin contraction (96). CalA treatment did not visibly alter actin cytoskeletal structure or cell viability. Inducing contraction with CalA decreased counts of PBs (Fig. 7D and E), again consistent with the hypothesis that actomyosin contractility controls PB disassembly.

Actomyosin contractility impacts cytoskeletal tension in adherent cells with SFs (97, 98). Additionally, both Jasp and CytD interfere with cytoskeletal tension (99), and both increased PB counts (Fig. 5A to C). Since the mechanoresponsive transcription activator, YAP, is activated by increases to cytoskeletal tension via actomyosin contractility (14), we explored the role of YAP in KapB-mediated PB disassembly.

### YAP is required for KapB-mediated PB disassembly.

**FIG 8:**
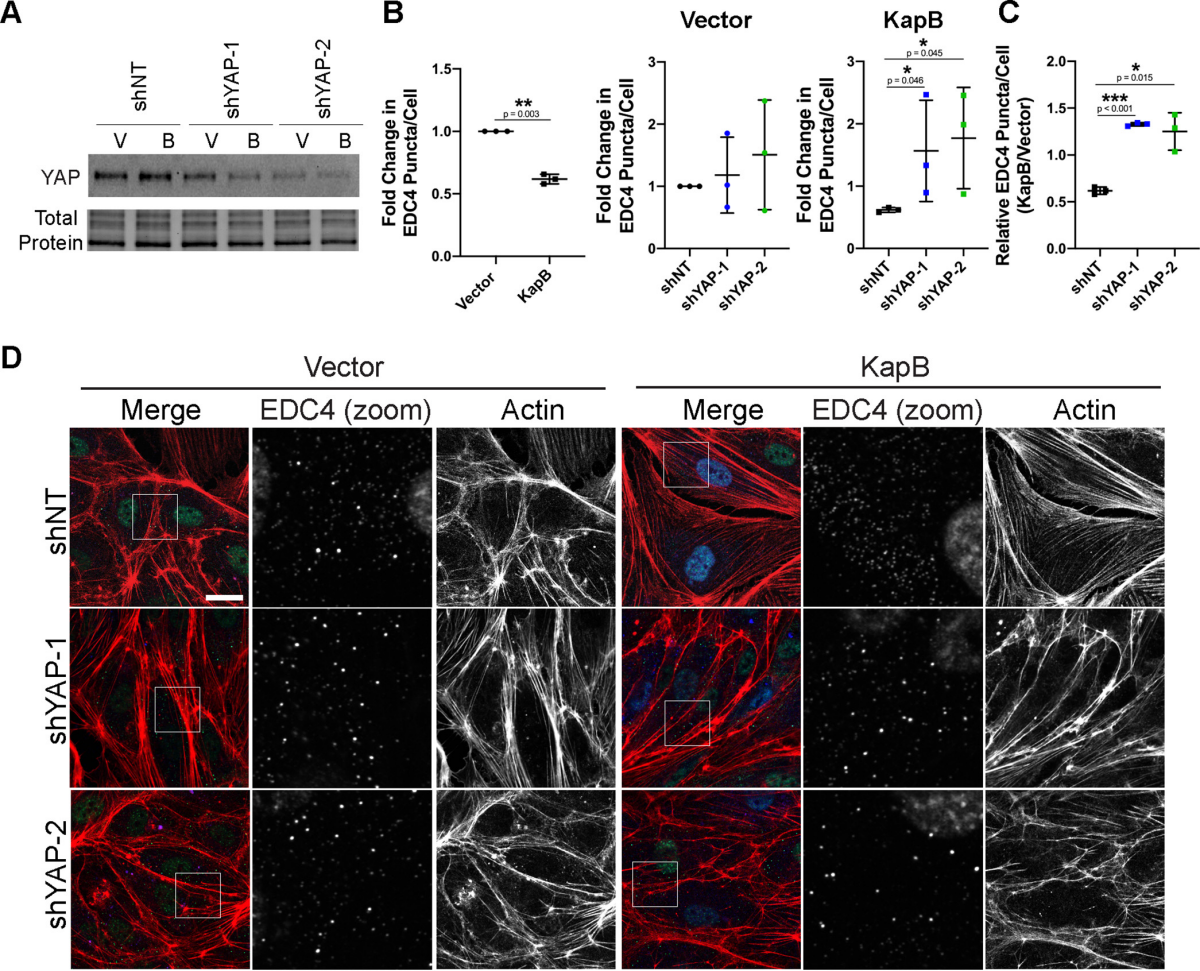
YAP is required for KapB-mediated PB disassembly. KapB- and vector-expressing HUVECs were transduced with shRNAs targeting YAP (shYAP-1 and shYAP-2) or with a nontargeting (shNT) control and selected. In parallel, cells were fixed for immunofluorescence or lysed for immunoblotting. (A) One representative immunoblot of three independent experiments stained with YAP-specific antibody is shown. (B to D) Fixed cells were stained for CellProfiler analysis as detailed in Materials and Methods. (B) The number of EDC4 puncta per cell was quantified and normalized to the vector NT control within each replicate. (C) CellProfiler data were used to calculate the ratio of EDC4 puncta count in KapB-expressing cells to the vector control for each treatment condition. (D) Representative images of cells stained for PB resident proteins EDC4 (green), KapB (blue), and F-actin (red, phalloidin). Boxes indicate area shown in the EDC4 (zoom) panel. Scale bar represents 20 μm. Statistics were determined using ratio-paired *t* tests between control and experimental groups; error bars represent standard deviation. *n* = 3 independent biological replicates. *, *P* < 0.05; **, *P* < 0.01; ***, *P* < 0.001.

To determine if YAP was involved in KapB-mediated PB disassembly, we expressed shRNAs targeting YAP in KapB-expressing HUVECs to assess whether the altered levels of YAP impacted PB disassembly. Immunoblotting confirmed knockdown efficiency of YAP shRNAs (Fig. 8A). Knockdown of YAP resulted in elongated HUVECs with mostly cortical actin fibers (Fig. 8D). These cells displayed increased PBs in KapB-expressing cells (Fig. 8B to D). In the context of YAP knockdown, the KapB/vector ratio of PBs counts was restored, indicating that YAP is required for KapB-mediated PB disassembly (Fig. 8C) and suggesting that KapB is activating a mechanoresponsive signaling axis to elicit PB disassembly via YAP.

**FIG 9:**
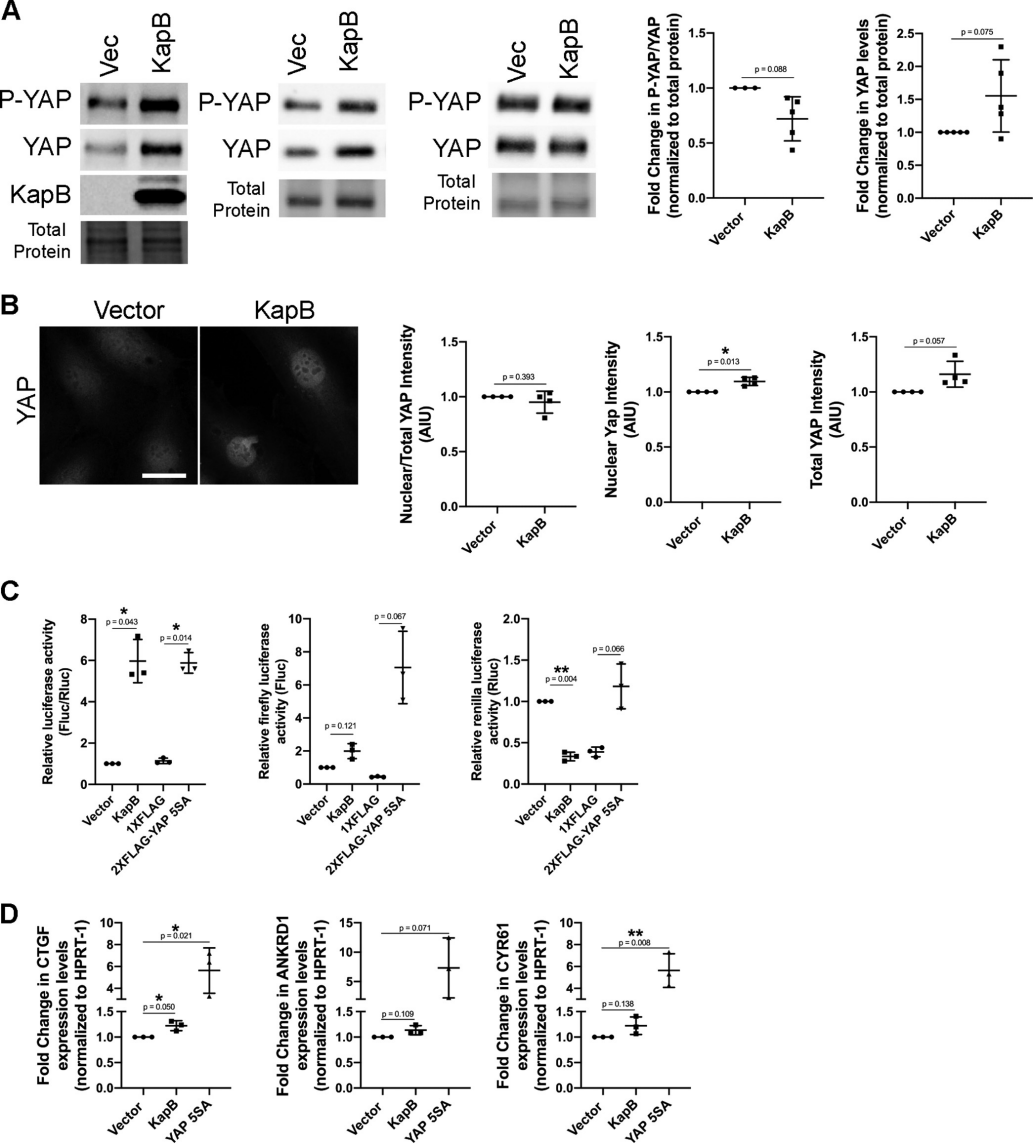
KapB does not activate canonical functions of YAP. (A and B) KapB- and vector-expressing HUVECs were lysed for immunoblotting or fixed for immunofluorescence. (A) Representative immunoblots and quantification of immunoblotting for P(S127)-YAP-, YAP-, or KapB-specific antibody are shown. Several immunoblots are shown to illustrate variation in KapB-mediated changes in P-YAP and YAP. Protein levels under each condition were normalized to total protein. All treatments were normalized to vector control within each replicate. (B) Representative images of cells stained for YAP. Scale bar represents 20 μM. (C) HEK-293A cells were cotransfected with a firefly luciferase (Fluc) reporter plasmid with a YAP-responsive TEAD promoter element, a TREX-*Renilla* luciferase (Rluc) reporter plasmid, and overexpression constructs for either a KapB, YAP 5SA, or vector control. At 36 h posttransfection, cells were starved in serum-free DMEM for 12 h and lysed, and Fluc and Rluc activity was recorded. Data are normalized to vector control. Graphs show the ratio of Fluc to Rluc, independent Fluc values, and independent Rluc values, respectively. (D) HUVECs were transduced with recombinant lentiviruses expressing KapB, a constitutively active version of YAP (YAP 5SA) or an empty vector control, selected, and lysed for total RNA. qRT-PCR analysis of steady-state mRNA levels of canonical YAP-regulated genes CTGF, ANKRD1 and CYR61 was performed and was normalized to steady-state HPRT-1 mRNA levels. Statistics were determined using repeated-measures ANOVA; error bars represent standard deviation. *n* = 3 independent biological replicates. *, *P* < 0.05; **, *P* < 0.01.

We next investigated the steady-state protein level and localization of YAP in KapB-expressing cells. KapB-transduced HUVECs showed increased levels of nuclear YAP and total YAP in some experiments, though the ratio of nuclear to cytoplasmic YAP was not markedly increased (Fig. 9A and B). When YAP is phosphorylated by large tumor suppressor kinase (LATS), it is sequestered in the cytoplasm and transcriptionally inactive (18). While YAP has multiple phosphorylation sites, phosphorylation at serine 127 is the most potent LATS-mediated phosphorylation site that promotes cytoplasmic distribution of YAP (18). To investigate the phosphorylation status of YAP in KapB-expressing cells, levels of P(S127)-YAP and total YAP were measured by immunoblotting. In KapB-expressing cells, there was a decrease in the ratio of P(S127)-YAP to total YAP that may suggest YAP is more active when KapB is expressed (Fig. 9A). In some experiments, we observed an increase in total steady-state levels of YAP by immunoblotting, corroborating the small increase in total YAP intensity seen by microscopy (Fig. 9A and B). Although increases in total YAP may suggest that YAP turnover is blocked or decreased by KapB, these increases are not accompanied by the expected strong decrease in phosphorylated YAP or an increase in percentage of total YAP found in the nucleus, as in Pavel et al. (100).

We next asked if active YAP can interact with TEAD and other transcription factors to elicit changes in gene expression in KapB-expressing cells (101). We used a TEAD element luciferase assay to assess if canonical YAP transcription was activated. As a positive control, we used YAP 5SA, a version of YAP that is unable to be phosphorylated and inactivated by the inhibitory kinase LATS (18) and is thus considered constitutively active. YAP 5SA robustly activated the TEAD element-containing firefly luciferase reporter (Fig. 9C). KapB did not induce the transcription of the TEAD element-containing firefly luciferase reporter (TEAD-Fluc) (Fig. 9C). Further, KapB did not increase steady-state mRNA levels of common YAP target genes CTGF, CYR61, and ANKRD1 by reverse transcriptase quantitative PCR (RT-qPCR), although these genes were elevated by YAP 5SA (Fig. 9D). These data indicate that despite the observation that YAP may be more abundant in KapB-expressing cells, canonical YAP targets are not upregulated.

**FIG 10:**
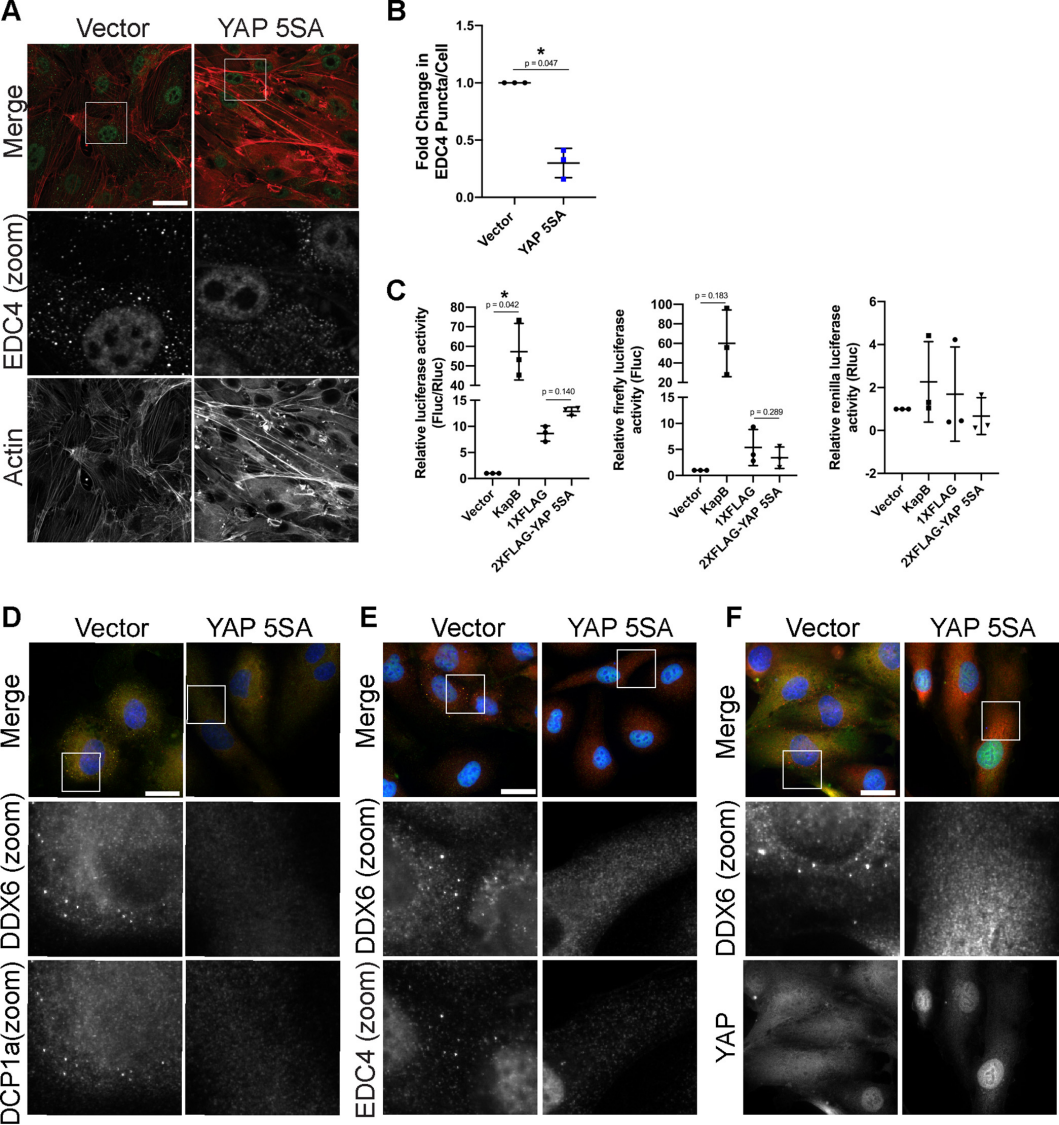
Active YAP elicits PB disassembly. (A, B, and D to F) HUVECs were transduced with YAP 5SA-expressing and empty vector lentivirus and selected. Cells were fixed for immunofluorescence. (A) Representative images of cells stained for PB resident proteins EDC4 (green) and F-actin (red, phalloidin). Boxes indicate area shown in the EDC4 (zoom) panel. Scale bar represents 20 μm. (B) Fixed cells were stained for CellProfiler analysis as detailed in Materials and Methods. The number of EDC4 puncta per cell was quantified and normalized to the vector control. (C) HeLa Tet-Off cells were seeded and cotransfected with an ARE-containing firefly luciferase (Fluc) reporter plasmid, a *Renilla* luciferase (Rluc) reporter plasmid lacking an ARE, and either a KapB, YAP 5SA expression plasmid, or vector controls. At 36 h posttransfection, transcription was terminated with doxycycline treatment for 12 h. Fluc and Rluc activity was measured. Data are normalized to vector control within each replicate. Graphs show the ratio of Fluc to Rluc, independent Fluc values, and independent Rluc values, respectively. (D to F) Representative images of cells stained for DDX6 (red), DCP1a (green), and DAPI (blue) (D); DDX6 (red), EDC4 (green), and DAPI (blue) (E); and DDX6 (red), YAP (green), and DAPI (blue) (F). Boxes indicate area shown in the zoom panels. Scale bar represents 20 μm. Statistics were determined using ratio-paired *t* tests between control and experimental groups (B) or repeated measures ANOVA (C); error bars represent standard deviation. *n* = 3 independent biological replicates. *, *P* < 0.05; **, *P* < 0.01.

We wondered if YAP activation could elicit PB disassembly in the absence of KapB expression. To this end, we transduced HUVECs with YAP 5SA. YAP 5SA-expressing cells contained few, thick actin fibers (Fig. 10A) and displayed decreased number of PBs per cell, indicating that YAP 5SA elicited disassembly of PBs (Fig. 10A and B). We also confirmed that we could stain PBs in control cells with antibodies for more than one PB resident protein (EDC4, Dcp1a, and DDX6) and that when YAP 5SA was expressed, loss of PB puncta was observed for each of these staining conditions (Fig. 10). KapB-mediated PB disassembly correlates with increases in stability and levels of ARE-mRNA (45, 73). To examine whether YAP 5SA-mediated PB disassembly elicits the same changes in ARE-mRNAs, we used a luciferase assay we previously established to measure the luminescence of an ARE-containing firefly luciferase reporter (102). In this assay, as previously shown in Corcoran et al. (45), KapB increased the level of firefly luminescence, indicating enhanced stability or translation of its RNA transcript (Fig. 10C). However, despite also inducing pronounced PB disassembly, YAP 5SA did not increase Fluc luminescence significantly more than the control construct (Fig. 10C). This points to a divergence of KapB and active YAP outcome. Although PB disassembly is induced by the expression of both YAP 5SA and KapB, active YAP increases the transcriptional activation of genes CTGF, CYR61, and ANKRD1, while KapB does not; conversely, KapB enhances the stability of ARE-mRNAs while active YAP does not.

**FIG 11:**
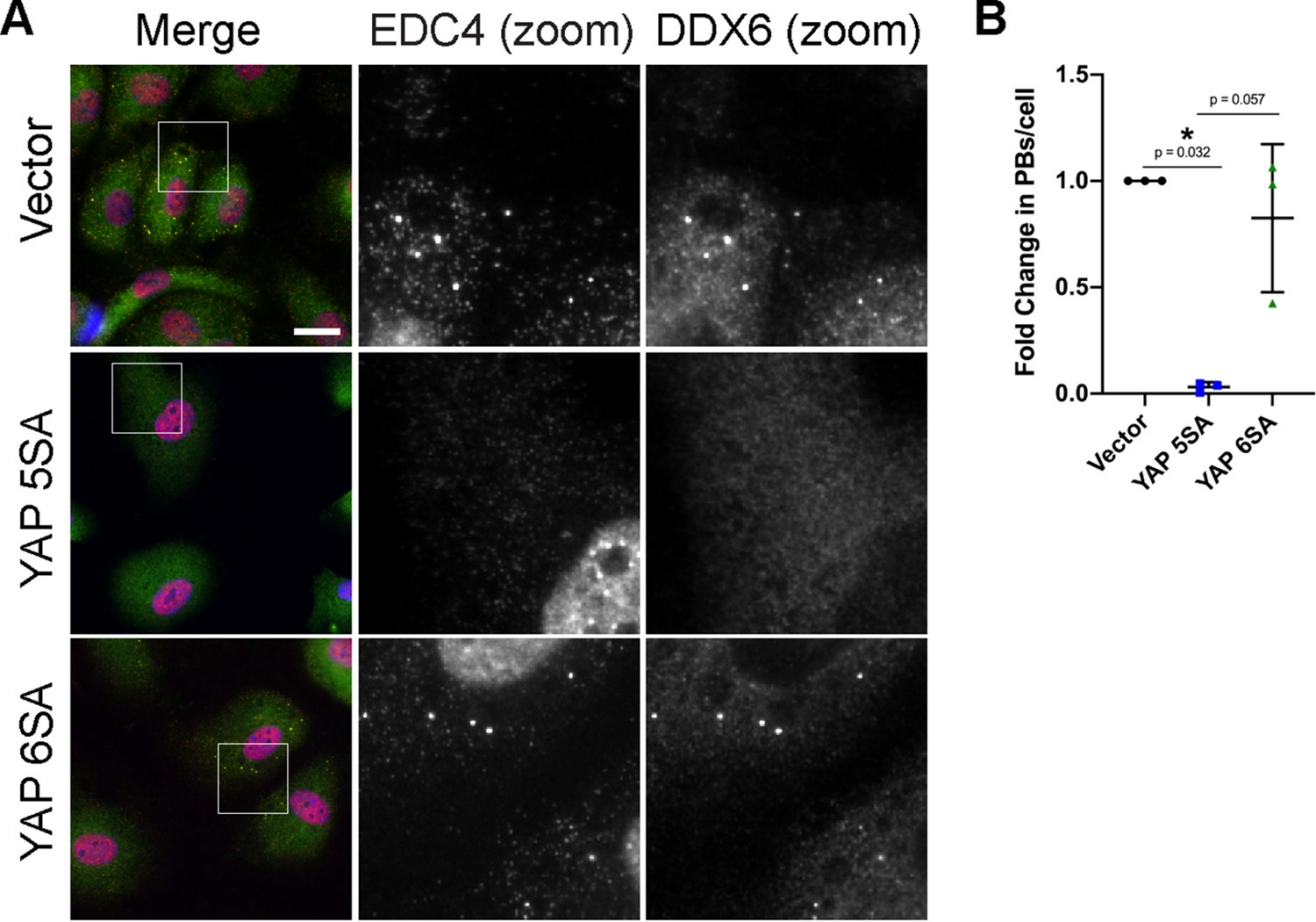
YAP transcriptional activity is required for PB disassembly. HUVECs were transduced with YAP 5SA-, YAP 6SA-, or vector-expressing lentivirus and selected. Cells were fixed for immunofluorescence. (A) Representative images of cells stained for EDC4 (red), DDX6 (green), and DAPI (blue). Boxes indicate area shown in the zoom panels. Scale bar represents 20 μm. (B) Fixed cells were stained for CellProfiler analysis as detailed in Materials and Methods. CellProfiler was used to count nuclei, EDC4 puncta, and DDX6 puncta. In RStudio analysis, puncta with ≥60% correlation (mean correlation in vector control) between EDC4 and DDX6 (PBs) were counted and normalized to number of nuclei per condition. PB counts were normalized to vector control within each replicate.

We wondered if PB disassembly did not require YAP to transactivate transcription of its canonical genes. To better understand this mechanism, we transduced HUVECs with another YAP construct called YAP 6SA (Fig. 11). YAP 6SA contains the same mutations as 5SA; in addition, it cannot be phosphorylated by AMPK at serine 94 (103). This phosphorylation event is essential for its interaction with TEAD; therefore, the S94A mutation renders YAP 6SA transcriptionally inactive (103, 104). We observed that, unlike YAP 5SA or KapB, YAP 6SA did not appear to elongate cells, suggesting that actin dynamics were unaffected, as in Pavel et al. (100). Compared to YAP 5SA, which caused pronounced PB disassembly, YAP 6SA failed to alter PB levels compared to vector controls (Fig. 11B). This was confirmed by staining PBs with antibodies for two different resident proteins, DDX6 and EDC4 (Fig. 11A).

### YAP activators disassemble PBs.

**FIG 12:**
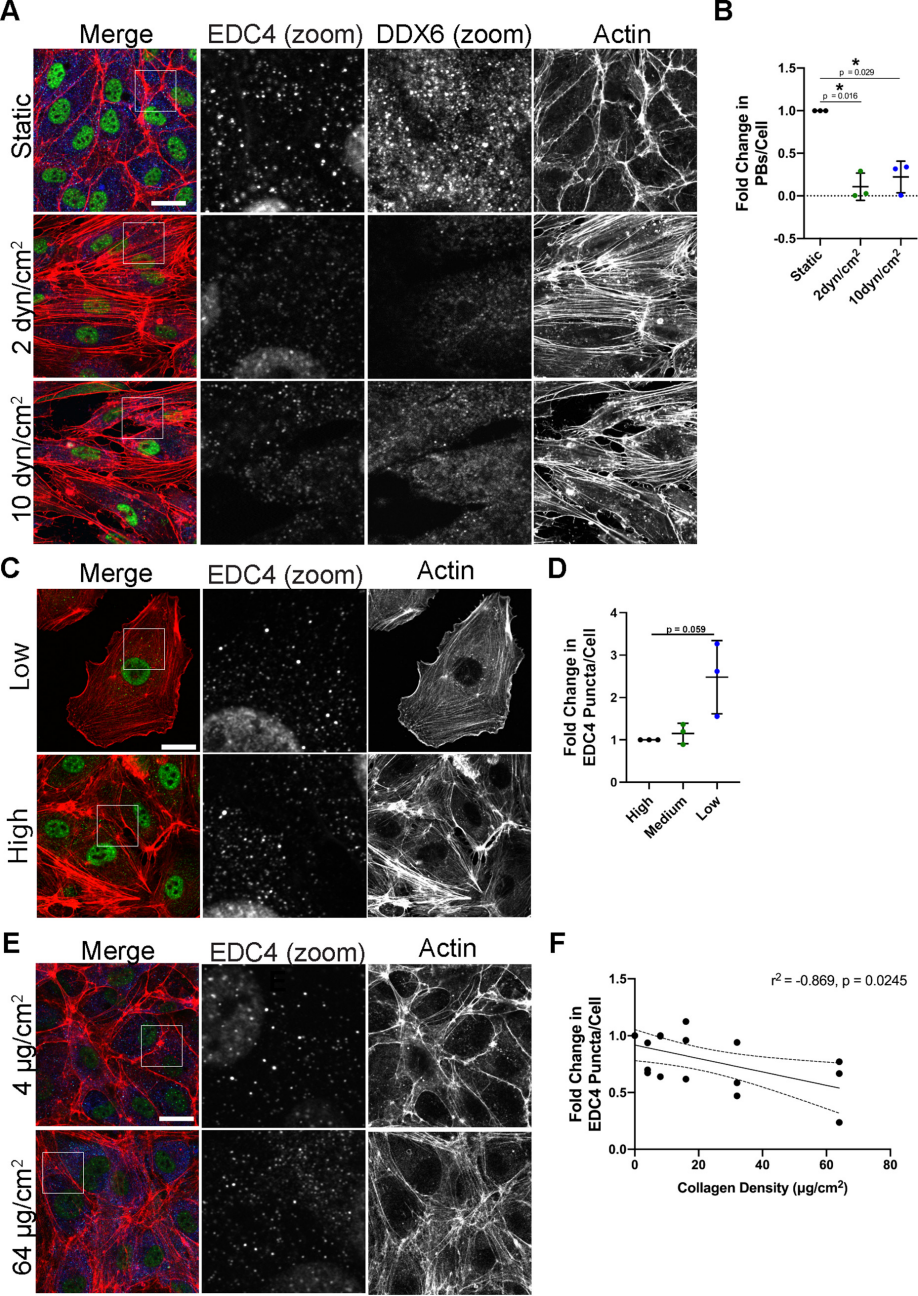
YAP inputs mediate PB disassembly. (A and B) HUVECs were seeded onto collagen-coated microscope slides and exposed to shear stress of 2 dyn/cm^2^, 10 dyn/cm^2^, or no shear (static control) for 21 h. Cells were fixed and stained for immunofluorescence. (A) Representative images of cells stained for PB resident proteins EDC4 (green) and DDX6 (blue), as well as F-actin (red, phalloidin). Boxes indicate area shown in EDC4 (zoom) and DDX6 (zoom) panels. Scale bar represents 20 μm. (B) CellProfiler was used to count nuclei, EDC4 puncta, and DDX6 puncta. In RStudio analysis, puncta with ≥70% correlation (mean correlation in vector control) between EDC4 and DDX6 (PBs) were counted and normalized to number of nuclei per condition. PB counts were normalized to static control within each replicate. (C and D) HUVECs were split and seeded at a high, medium, and low density, cultured for 48 h, and fixed for immunofluorescence. (C) Representative images of cells stained for the PB resident proteins EDC4 (green) and F-actin (red, phalloidin). Boxes indicate images shown in EDC4 (zoom) panel. Scale bar represents 20 μm. (D) Fixed cells were stained for CellProfiler analysis as detailed in Materials and Methods. The number of EDC4 puncta per cell was quantified and normalized to the high confluence condition. (E and F) Coverslips were coated for 4 h with 0 to 64 μg/cm^2^ of collagen. HUVECs were grown for 72 h on coated coverslips and fixed for immunofluorescence. (E) Representative images of cells stained for PB resident proteins EDC4 (green), DDX6 (blue), and F-actin (red, phalloidin). Boxes indicate images shown in EDC4 (zoom) panel. Scale bar represents 20 μm. (F) Fixed cells were stained for CellProfiler analysis as detailed in Materials and Methods. The number of EDC4 puncta per cell was quantified and normalized to the 0 μg/ml collagen-coating condition. Statistics were determined using repeated-measures ANOVA (A and B) or Pearson’s correlation coefficient (C); error bars represent standard deviation (A and B) and 95% confidence interval of line of best fit (slope is significantly nonzero; *P* = 0.014) (C). *n* = 3 independent biological replicates. *, *P* < 0.05; **, *P* < 0.01.

Since overexpression of constitutively active YAP 5SA leads to disassembly of PBs, we wanted to determine whether other stimuli that activated endogenous YAP could do the same. We tested various upstream mechanical signals described to activate YAP for their ability to elicit PB disassembly, including shear stress, low cell confluence, and high ECM stiffness (14, 18, 20, 22, 24). For the first, we subjected HUVECs to shear stress by fluid flow (shear forces of 2 and 10 dyn/cm^2^), and PBs were examined via immunofluorescence using antibodies to both EDC4 and DDX6. Both treatments showed prominent cell elongation and stress fiber formation and resulted in robust PB disassembly of both EDC4-positive and DDX6-positive puncta (Fig. 12A and B). To test if cell confluence regulates PB levels, HUVECs were seeded at low, medium, and high densities. Cells at low confluence are reported to have active YAP, and we predicted PBs would disassemble; however, the low-density monolayer displayed more PBs per cell than those at medium and high densities (Fig. 12C and D). To test the impact of collagen stiffness on PB disassembly, HUVECs were plated on coverslips coated with increasing densities of collagen (0 to 64 μg/cm^2^). While collagen density does not perfectly reproduce matrix stiffness, as it does not differentiate the effect of matrix stiffness from increasing collagen-binding sites, increasing collagen densities do correlate with increased matrix stiffness (105–107). As collagen density increased, PBs decreased (Fig. 12E and F). Notably, as collagen density increased, only slight increases in visible actin fibers were noted (Fig. 12E), suggesting the tested range of stiffness was small and should be expanded in future studies. Taken together, these data indicate that PB disassembly occurred in response to mechanical stimuli known to require RhoA and altered cytoskeletal structures to activate YAP (shear stress and increased ECM concentration) (23, 25, 108, 109). Again, our model points to the importance of actin SF formation as a requisite precursor to PB disassembly, irrespective of YAP activation status.

### Shear stress-mediated PB disassembly requires YAP.

**FIG 13:**
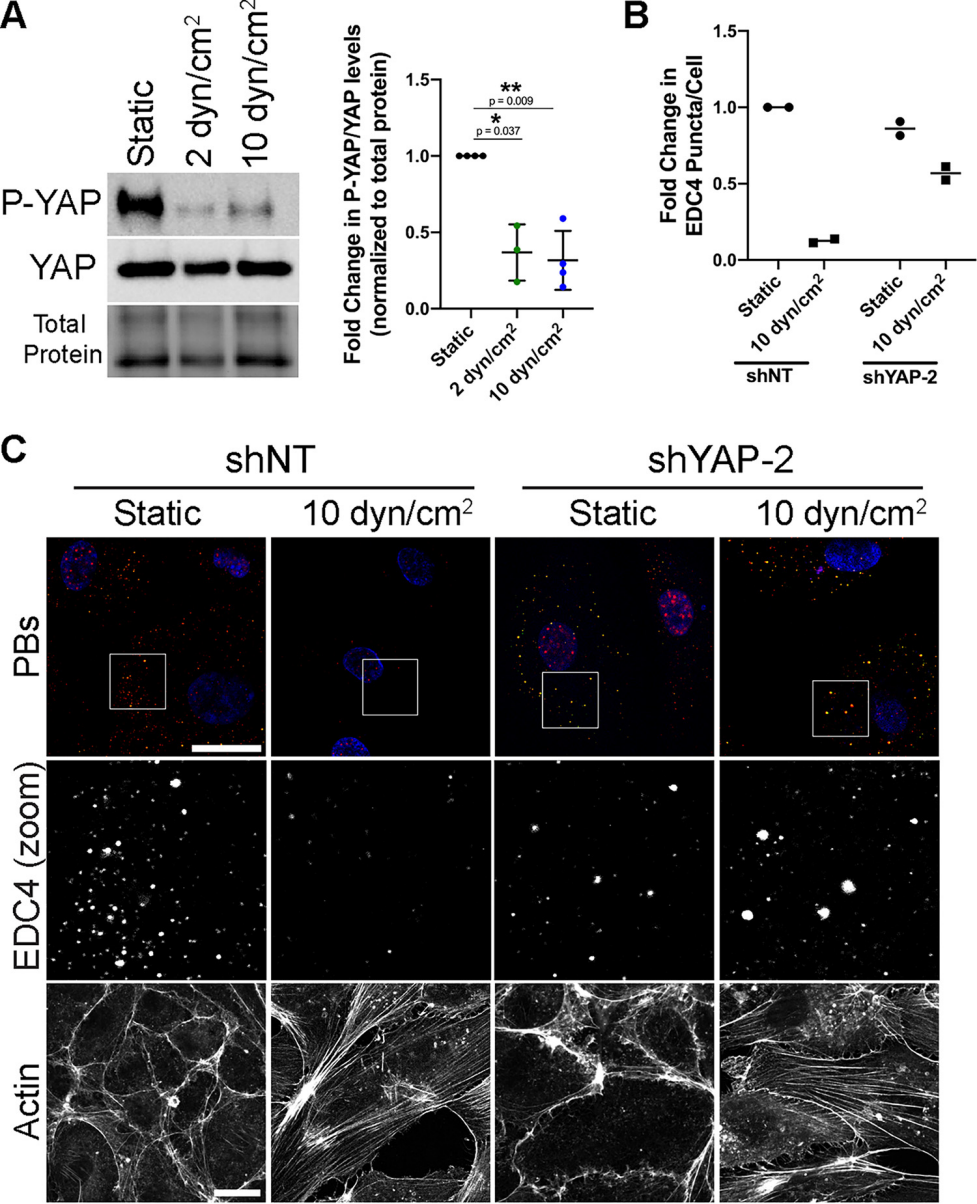
YAP is required for EDC4 puncta disassembly in HUVECs subjected to shear stress. (A) HUVECs were seeded onto collagen-coated microscope slides and exposed to shear stress of 2 dyn/cm^2^, 10 dyn/cm^2^, or a static control for 21 h. Cells were lysed for immunoblotting. One representative immunoblot and quantification of three independent experiments stained with P(S127)-YAP- and YAP-specific antibody are shown. P(S127)-YAP and YAP protein levels under each condition were normalized to total protein. All treatments were normalized to static control within each replicate. (B and C) HUVECs were transduced with shRNAs targeting YAP (shYAP-2) or with a nontargeting (shNT) control and selected. Cells were seeded onto collagen-coated microscope slides and exposed to shear stress of 10 dyn/cm^2^ or no shear (static control) for 21 h. Cells were fixed and stained for immunofluorescence. (B) CellProfiler was used to count nuclei and EDC4 puncta. In RStudio analysis, EDC4 puncta were normalized to number of nuclei per condition. EDC4 puncta counts were normalized to static control. (C) Representative images of cells stained for PB resident proteins EDC4 (red), DDX6 (green), and DAPI (blue). In parallel, separate coverslips were stained for F-actin (phalloidin). Boxes indicate area shown in the EDC4 (zoom) panel. Scale bar represents 20 μm. Statistics were determined using repeated-measures ANOVA (A); error bars represent standard deviation (A). *n* = 4, except 2 dyn/cm^2^ (*n* = 3) (A) and *n* = 2 (B and C) independent biological replicates. *, *P* < 0.05; **, *P* < 0.01.

YAP responds to external forces that induce active RhoA, actin SFs, and pronounced cell elongation, in short, the typical behavior of ECs in response to the mechanical shear stress that is produced by fluid flow. However, how YAP responds to shear stress is controversial (19, 22, 23, 110). To verify YAP activation by continuous, unidirectional fluid flow in our system, HUVECs subjected to 2 and 10 dyn/cm^2^ of shear stress were lysed and used for immunoblotting for P(S127)-YAP and total YAP. Shear stress decreased the ratio of phospho-YAP/YAP in both conditions, suggesting a higher proportion of active YAP (Fig. 13A). To assess if YAP was required for PB disassembly in response to shear stress, HUVECs transduced with YAP-targeting shRNA were subjected to 10 dyn/cm^2^ shear stress. PBs disassembled in cells treated with a nontargeting shRNA when subjected to shear stress (Fig. 13B and C), consistent with earlier experiments (Fig. 13A and B). When YAP was reduced by shRNA expression, ECs exposed to shear stress had more PBs than shear-treated shNT control cells (Fig. 13B and C). YAP knockdown also reduced the cells’ ability to form SFs, with many cells displaying highly cortical phalloidin staining and fewer actin fibers across the cell (Fig. 13C). Therefore, YAP is required to disassemble PBs after KapB is expressed and also in response to shear stress.

Recently, an important connection has emerged between YAP and autophagy. Active YAP was previously shown to upregulate the transcription of Armus, a Rab7-GTPase-activating protein required for autophagosome fusion with the lysosome, the final step in the autophagic pathway (111, 112). Moreover, stimuli that promote active YAP activate autophagic flux by inducing the transcription of TEAD-responsive genes that regulate actomyosin, including ACTNB and MYOII (100). These data showed that actin and myosin modulate the trafficking of key autophagy proteins (such as Atg16L1 and Atg9A) that are required for autophagosome formation and that this process is a downstream effect of YAP activation and YAP 5SA (100).

**FIG 14:**
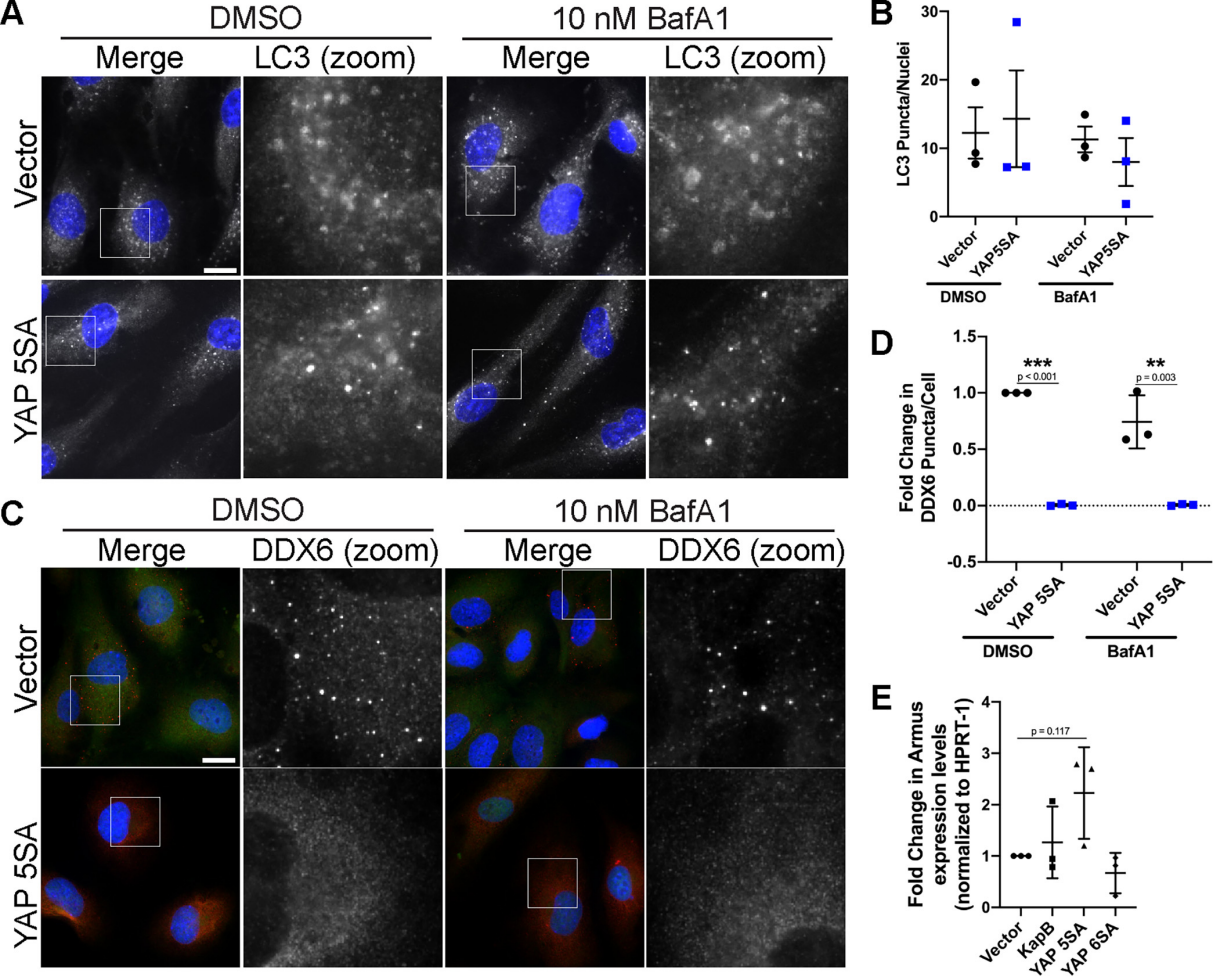
YAP 5SA-mediated PB disassembly is not dependent on autophagy. YAP 5SA- and vector- expressing HUVECs were treated with 10 nM BafA1 or DMSO control for 30 min and fixed for immunofluorescence. (A and B) Fixed cells were stained for LC3 puncta analysis as detailed in Materials and Methods. (A) Representative images of cells stained for LC3 (white) and DAPI (blue). Boxes indicate the area of the field of view that is shown in LC3 (zoom) panel. Scale bar represents 20 μm. (B) The number of LC3 puncta per cell was quantified under each condition. (C and D) Fixed cells were stained for CellProfiler analysis as detailed in Materials and Methods. (C) Representative images of cells stained for DDX6 (red), YAP (green) and DAPI (blue). Boxes indicate the area of the field of view that is shown in DDX6 (zoom) panel. Scale bar represents 20 μm. (D) The number of DDX6 puncta per cell was quantified and normalized to the vector control within each replicate. (E) HUVECs were transduced with recombinant lentiviruses expressing KapB, a constitutively active version of YAP (YAP 5SA) or an empty vector control, selected, and lysed for total RNA. qRT-PCR analysis of steady-state mRNA levels of the Rab7-GTPase-activating protein, Armus, was performed and was normalized to steady-state HPRT-1 mRNA levels. Statistics were determined using a two-way ANOVA with multiple comparisons between control and experimental groups; error bars represent standard deviation. *n* = 3 independent biological replicates. **, *P* < 0.01; ***, *P* < 0.001.

To examine the connection between YAP and autophagic flux as it pertains to PB disassembly, we used immunofluorescence to stain for the autophagy marker, LC3, which forms puncta that represent autophagosomes (Fig. 14). YAP5SA-expressing cells did not have more LC3 puncta than vector controls, and LC3 puncta did not increase after treatment with bafilomycin A1 (an inhibitor of lysosomal acidification and autophagosome-lysosome fusion) (113) (Fig. 14). The lack of LC3 puncta increase in YAP 5SA-expressing cells after bafilomycin A1 treatment could mean that autophagy is not increased. It could also mean that autophagic flux and degradation are both increased, LC3 is degraded rapidly, and LC3 puncta fail to accumulate. The latter interpretation is most consistent with previous reports which showed that YAP 5SA enhances autophagic flux (100) and promotes autophagosome-lysosome fusion (111). We also observed that YAP 5SA increased the transcription of Armus, involved in activation of Rab7 for autophagosome-lysosome fusion, while KapB did not (Fig. 14). To determine if enhanced autophagic flux and accelerated autophagosome turnover caused by YAP 5SA contributes to PB disassembly, we treated YAP 5SA-expressing cells with bafilomycin A1 to block the final autophagy step and stained for PBs (Fig. 14). We observed that bafilomycin A1 did not restore PBs in YAP 5SA-expressing cells (Fig. 14). This suggests the possibility that autophagy is not the primary mechanism required for PB disassembly induced by YAP 5SA and that other inputs or factors are also involved.

Taken together with our analysis of KapB-mediated PB disassembly, these data suggest that when KapB is expressed, it turns on mechanoresponsive signals that endothelial cells use to withstand mechanical forces like shear, in the absence of an external stimulus. The outcome of both scenarios is YAP-dependent disassembly of cytoplasmic PBs.

## DISCUSSION

In this paper, we have used a viral protein from an oncogenic virus to uncover the relationship between cytoplasmic PBs and the mechanical regulation of actin SF formation. We present data to support the existence of a mechanoresponsive pathway that links actin SFs, actomyosin contractility, and the transcription transactivator YAP to the disassembly of PBs and show that this pathway is hijacked by KapB during KSHV latency. Our major findings are as follows: (i) KapB-mediated PB disassembly requires actin SF effectors ROCK1/2/mDia1 and is enhanced by loss of the actin-severing protein, cofilin; (ii) KapB-mediated PB disassembly is reversed when blebbistatin is used to inhibit actomyosin contractility or after knockdown of the mechanoresponsive transcription transactivator, YAP; (iii) in the absence of KapB, we can induce PB disassembly when we promote the formation of actin SFs, actomyosin contractility, and YAP activity using overexpression of α-actinin-1 (promotes actin bundling into SFs and increases cytoskeletal tension) (93), calyculin A (inhibits myosin light chain phosphatase to promote actomyosin contraction) (96), or active YAP (YAP 5SA). Exposure of endothelial cells to the external forces created by shear stress or a stiff extracellular matrix also induces PB disassembly in the absence of KapB; and (iv) overexpression of transcriptionally inactive YAP (YAP 6SA) fails to disassemble PBs, indicating that YAP’s role as a transcription transactivator is important for its ability to promote PB disassembly. Together, these data show, for the first time, that PBs disassemble in response to mechanical signals that transduce external forces from outside the cell to the actin cytoskeleton and that this is a pathway used by endothelial cells to regulate gene expression in response to diverse stimuli. Moreover, this work also highlights the remarkable pizzazz used by viruses to hijack cellular pathways. In this case, we reveal that the viral protein KapB taps into this mechanoresponsive pathway to trigger mechanical changes to cytoskeletal structures and downstream effectors that would normally respond to force, thereby inducing PB disassembly from within the cell, rather than from without.

During the process of actin polymerization, the monomeric form of actin, globular actin (G-actin), aggregates in groups of three subunits or more to nucleate an actin filament, which extends into filaments via addition of further G-actin monomers through ATP-dependent polymerization (114). Ten to 30 actin filaments (F-actin) bundle together into SFs, primarily using the α-actinin family for cross-linking (88, 115–117). SFs with periodic distribution of actin-cross-linking proteins and nonmuscle myosin II (NMII) are contractile structures (97, 98), but not all actin SFs function equally in this regard. For any structure to be able to generate tension, it must be tethered at the ends. Of the types of SFs (ventral SFs, dorsal SFs, and transverse arcs [115]), ventral SFs are attached at both termini to the extracellular matrix (ECM) through focal adhesions and contain NMII, which imparts a contractile phenotype (6, 76, 115). Dorsal SFs are attached through focal adhesions but do not contain NMII and thus are not contractile (6, 115). However, dorsal SFs are thought to work in concert with transverse arcs, which contain NMII but are not attached to focal adhesions, to mediate cellular contractility.

Several features of our data suggest that the structures that are important for KapB-mediated PB disassembly must be contractile and cause cytoskeletal tension. When both mDia1 and ROCK2 were silenced in KapB-expressing cells (Fig. 1 and 2), visible actin bundles are still apparent despite PB restoration in both contexts. This suggests that not all SF subtypes are required for our phenotype. In addition, blebbistatin treatment of KapB-expressing cells dramatically restored PBs; these data suggest that PB disassembly requires actin-mediated contractility rather than mere structural support (Fig. 7). Furthermore, overexpression of α-actinin and shear stress increase cell stiffness (93, 118), and both treatments induced PB disassembly (Fig. 6 and 12), reinforcing the correlation between increasing cell tension and PB disassembly. Finally, our data show that YAP is required for PB disassembly (Fig. 8, 10, and 13). YAP is mechanoresponsive; it becomes active when tension-forming actin structures are induced by external forces, e.g., focal adhesion engagement by stiff ECM (14, 119). As YAP activation and PB disassembly both rely on RhoA-induced cytoskeletal contractility, any activator of YAP that induces cytoskeletal tension through RhoA should mediate PB disassembly. Our data support this notion, as shear stress forces and increasing collagen density both cause PB disassembly in the absence of KapB, while low confluence does not (Fig. 12). We also know that G protein-coupled receptors (G_11/12_ and G_q/11_) activate YAP in a RhoA-dependent manner (17), and LPA treatment or overexpression of KSHV-derived constitutively active vGPCR (both activate G_11/12_) both induce PB disassembly (43, 71). These findings support the conclusion that PB disassembly requires the formation of contractile actin structures like those associated with YAP transactivation responses.

KSHV is an oncogenic virus associated with the endothelial neoplasm, Kaposi’s sarcoma (KS). Cells within the KS lesion display latent KSHV infection, proliferate abnormally, spindle, and release many proinflammatory and protumorigenic mediators into the microenvironment. KapB expression alone recapitulates two of these key features, cell spindling and proinflammatory mediator production that results from enhanced stability of ARE-containing cytokine mRNAs that would normally shuttle to PBs for constitutive turnover (45). Our previous work showed that both phenotypes utilize KapB activation of the stress-responsive kinase, MK2, and the downstream activation of the GTPase RhoA (45, 71). We also showed that the lytic vGPCR protein mediates PB disassembly and the concomitant stabilization of ARE-mRNAs, while more recently, open reading frame 57 (ORF57) has been also reported to disrupt PBs (43, 120). The observation that KSHV encodes at least three separate gene products sufficient to drive PB disassembly suggests that PB disassembly is beneficial for some aspect of the infectious cycle. Further research is required to definitively address how PBs influence the KSHV infectious cycle and the fate of infected cells.

We and others observed that the presence or absence of PB punctae visible by microscopy directly correlates with the stability of ARE-mRNAs (45, 57, 58). We predicted that YAP-mediated PB disassembly would also promote ARE-mRNA stability. Indeed, several YAP target genes contain ARE elements in their 3′ UTRs, including CTGF and ANKRD1 (65, 121). Shear forces also cause YAP-dependent PB disassembly and have previously been shown to upregulate many genes containing ARE-mRNAs (65, 122). However, in our studies, overexpression of constitutively active YAP (YAP 5SA) disassembled PBs but did not increase stability of an ARE-containing firefly luciferase reporter (Fig. 10) (102). This may be due to different functional responses for different classes of AU-rich elements. Our ARE-containing luciferase reporter contains the AU-rich sequence derived from the 3′ UTR of granulocyte-macrophage colony-stimulating factor (GM-CSF), categorized in cluster 5, whereas canonical YAP genes CTGF and ANKDR1 are in clusters 1 and 2, respectively (65). This discrepancy may also result from the dual function of PBs as sites of translational suppression as well as RNA decay, supported by observations that PBs decreases do not always correlate with decreased mRNA turnover (51–55).

**FIG 15:**
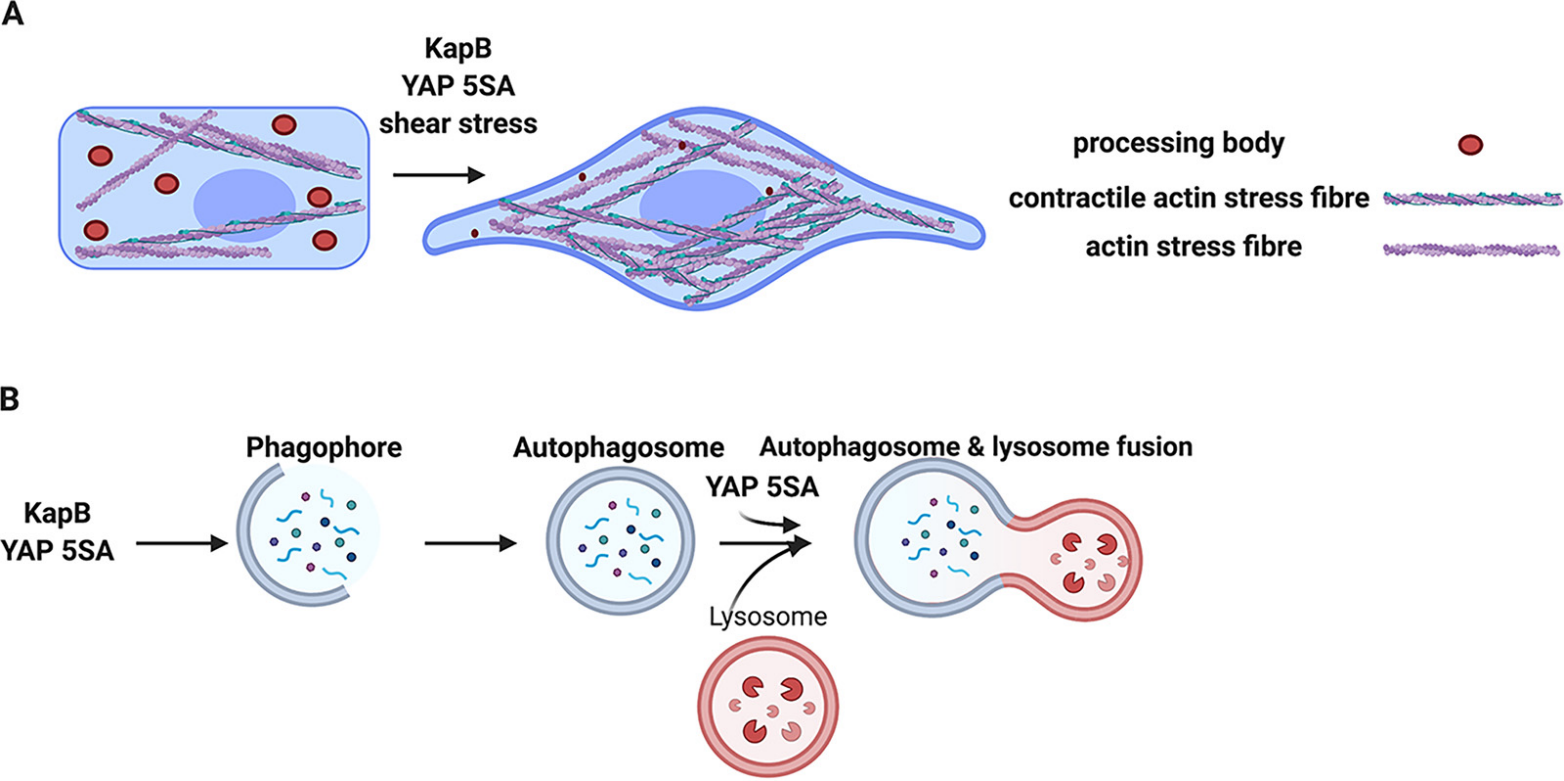
KapB activates a mechanoresponsive pathway from within the cell rather than without to mediate PB disassembly. (A) Cells respond to external mechanical force by activating their structural support network, the actin cytoskeleton. The GTPase RhoA and its downstream effectors coordinate this response, bundling actin filaments into stress fibers (SFs), enhancing actomyosin contractility, and increasing adhesion to the underlying matrix to help withstand force-induced membrane deformation. Together, these actin-based responses increase cytoskeletal tension and elicit the dephosphorylation and nuclear translocation of the mechanoresponsive transcription activator YAP, where it collaborates with other transcription factors to induce TEAD-responsive genes. We present data to support the existence of a mechanoresponsive pathway that links actin SFs, actomyosin contractility, and the transcription transactivator YAP to the disassembly of PBs. The viral protein KapB taps into this mechanoresponsive pathway, triggering mechanical changes and forming contractile cytoskeletal structures that would normally respond to force, thereby inducing PB disassembly in a YAP-dependent manner from within the cell, rather than from without. Both KapB and stimuli that activate YAP cause PB disassembly. (B) Both KapB and active YAP have been shown to upregulate autophagic flux (100, 131); however, YAP 5SA also accelerates autophagosome-lysosome fusion by upregulating the Rab7-GTPase-activating protein, Armus, while KapB does not increase Armus transcription. In our model, upregulated autophagic flux contributes to PB disassembly mediated by both YAP 5SA and KapB.

Data presented herein clearly implicate a requirement for YAP in the PB disassembly phenotype that is induced by KapB and by the external force, shear stress (Fig. 8 and 13). However, the precise connection between YAP and PB disassembly is unclear. What we do know is that despite the clear reliance on YAP for PB disassembly, KapB does not increase expression of canonical YAP-regulated transcripts (Fig. 9). Our data also show small increases in total YAP and small decreases in the ratio of phosphorylated/total YAP; however, the ratio of nuclear to cytoplasmic YAP is not markedly altered (Fig. 9). Taken together, these data suggested a model whereby PB disassembly was independent of YAP’s role as a gene transactivator and YAP nuclear translocation. However, we found that overexpression of YAP 6SA, which is unable to act as a transcription transactivator, fails to cause PB disassembly (Fig. 11), indicating that this transcription induction function of YAP is required for its role in regulating PB dynamics. In the discussion that follows, we describe our working model (Fig. 15) for how YAP may promote PB disassembly.

Several studies link YAP with the regulation of the catabolic process of autophagy, though many of these are contradictory and suggest YAP-mediated autophagy control is cell type and context dependent (100, 111, 123–125). Totaro et al. provided evidence that YAP promotes autophagic catabolism by promoting the expression of Armus, a Rab7-GTPase-activating protein that is required to mediate the fusion of autophagosomes with lysosomes in the final degradative step of autophagy (111). In addition, stimuli that activate YAP promote autophagic flux by inducing the transcription of TEAD-responsive genes that regulate actomyosin, including ACTNB and MYOII (100). These data showed that actin and myosin modulate the trafficking of key autophagy proteins (such as Atg16L1 and Atg9A) that are required for autophagosome formation and that this process is a downstream effect of YAP activation (100). These data also show that YAP 5SA promotes autophagic flux, while YAP 6SA does not, consistent with YAP-dependent autophagy upregulation requiring YAP transcriptional responses. This emerging connection between YAP and autophagy suggests that the latter is a mechanoresponsive process; this is also supported by other studies wherein endothelium exposed to unidirectional shear stress upregulates autophagy (126–130). These data are also consistent with new data from our group showing that PB disassembly mediated by KapB requires autophagy, as knockdown of a canonical autophagy gene (Atg5) or treatment with bafilomycin A1 restored PBs (131). However, YAP 5SA-mediated PB disassembly was not restored by bafilomycin A1 treatment (Fig. 14). This difference may be due, in part, to the higher levels of Armus expression in YAP 5SA-expressing cells than in KapB-expressing cells (Fig. 14). This would imply that although both YAP 5SA and KapB enhance autophagic flux, only YAP 5SA also accelerates the final degradative step of autophagy (Fig. 15).

PBs are sites where innate immune factors congregate that are disrupted by most viruses during infection (132–141). Indeed, KSHV encodes three separate proteins that all induce PB disassembly, suggesting this event is central to viral persistence (43, 45, 120). PBs are likely playing an as-yet-undefined and underappreciated role in regulating innate antiviral responses. YAP is also an unappreciated negative regulator of innate immune signaling pathways. YAP blocks the ability of the innate immune kinase, TBK1, a downstream effector for several innate signaling pathways, to associate and activate its substrates (104). In so doing, YAP blocks downstream induction of interferons and increases viral replication (104). However, this feature of YAP is independent of its ability to act as a transcriptional transactivator (104). Based on our data that show YAP 5SA causes PB disassembly while YAP 6SA does not, we think it most likely that KapB-induced PB disassembly requires the transcription transactivation function of YAP. We also speculate that KapB-induced PB disassembly, like active YAP, favors viral replication and survival and is promoted by KSHV in order to reshape subsequent antiviral innate immune responses.

In this paper, we describe the surprising convergence of two previously unrelated yet essential regulators of cellular gene expression—the mechanoresponsive transactivator YAP and cytoplasmic PBs, known sites of AU-rich cytokine mRNA decay. We show that PB disassembly is mechanoresponsive; external forces that change cell shape and tension-forming cytoskeletal structures cause PB disassembly in a YAP-dependent manner. This discovery was made courtesy of the unique KSHV protein, KapB, and provides yet another example of how viruses have evolved surprising ways to manipulate their host and ensure their survival. In this case, KapB induces, from the inside out, a mechanoresponsive pathway to cause PB disassembly. Future study will untangle how these related mechanoresponsive events are induced by KSHV to better promote viral replication.

## MATERIALS AND METHODS

### Antibodies, plasmids, and reagents.

The antibodies used in this study can be found in Table 1. The plasmids used in this study can be found in Table 2. Forward and reverse shRNA sequences were selected from the TRC Library Database in the Broad Institute RNAi Consortium. YAP target shRNAs in pLKO.1 were obtained from C. McCormick (Dalhousie University, Halifax, Canada). Sequences for all shRNA oligonucleotides used for cloning are listed in Table 3. Cloning of shRNAs was conducted according to the pLKO.1 protocol (142). YAP 6SA was subcloned into pLJM1 by PCR amplification using primers (forward, 5′-CGTAACCGGTATGGATCCCGGGCA-3′; reverse, 5′-CTGATAAGTCGACAACCACTTTGTACAAGAAAGTTG-3′) designed to remove the V5 tag and insert a stop codon as well as 5′ AgeI and 3′ SalI restriction enzyme sites. The chemical inhibitors used in this study can be found in Table 4.

**TABLE 1:** Antibodies used in this study

Antibody^*a*^	Source (catalog no.)	Use	Dilution
Rabbit anti-mDia1	Cell Signaling Technologies (5486)	Immunoblotting	1:1,000 in 2.5% BSA
Rabbit anti-ROCK1	Cell Signaling Technology (4035)	Immunoblotting	1:1,000 in 2.5% BSA
Rabbit anti-ROCK2	Cell Signaling Technology (9029)	Immunoblotting	1:500 in 2.5% BSA
Rabbit anticofilin	Cell Signaling Technology (5175)	Immunoblotting	1:1,000 in 2.5% BSA
Rabbit anti-P-YAP	Cell Signaling Technology (4911)	Immunoblotting	1:1,000 in 2.5% BSA
Rabbit anti-YAP	Cell Signaling Technology (4912)	Immunoblotting	1:1,000 in 2.5% BSA
Anti-mouse IgG, HRP linked (2°)	Cell Signaling Technology (7076)	Immunoblotting	1:2,000 to 1:4,000 in 2.5% BSA
Anti-rabbit IgG, HRP-linked (2°)	Cell Signaling Technology (7074)	Immunoblotting	1:2,000 to 1:4,000 in 2.5% BSA
Mouse anti-p70 s6 kinase (detects EDC4)	Santa Cruz (sc-8418)	Immunofluorescence	1:1,000 in blocking buffer (1% human AB in PBS), 4°C overnight
Rabbit anti-KapB	Gift from D. Ganem and C. McCormick	Immunofluorescence/immunoblotting	1:1,000 in blocking buffer (1% human AB in PBS), 30 min RT
Rabbit anti-actinin-1	ABclonal (A1160)	Immunofluorescence	1:500 in blocking buffer (1% human AB in PBS), 4°C overnight
Mouse anti-actinin-4	Santa Cruz (sc-390205)	Immunofluorescence	1:500 in blocking buffer (1% human AB in PBS), 4°C overnight
Mouse anti-YAP	Santa Cruz (sc-101199)	Immunofluorescence	1:1,000 in blocking buffer (1% human AB in PBS), 4°C overnight
Rabbit anti-DDX6	Bethyl Labs (A300-461A)	Immunofluorescence	1:1,000 in blocking buffer (1% human AB in PBS), 4°C overnight
Rabbit anti-β-actin, HRP linked	Cell Signaling Technology (5125)	Immunoblotting	1:2,000 in 2.5% BSA
Rabbit anti-LC3B	Cell Signaling Technology (2775)	Immunofluorescence	1:200 in blocking buffer (1% human AB in PBS), 4°C overnight
Alexa Fluor 555-conjugated donkey anti-mouse IgG (2°)	Invitrogen (A31570)	Immunofluorescence	1:1,000 in blocking buffer (1% human AB in PBS), 1 h RT
Alexa Fluor 488-conjugated chicken anti-rabbit IgG (2°)	Invitrogen (A21441)	Immunofluorescence	1:1,000 in blocking buffer (1% human AB in PBS), 1 h RT
Alexa Fluor 555-conjugated donkey anti-rabbit IgG (2°)	Invitrogen (A31572)	Immunofluorescence	1:1,000 in blocking buffer (1% human AB in PBS), 1 h RT
Alexa Fluor 488-conjugated chicken anti-mouse IgG (2°)	Invitrogen (A21200)	Immunofluorescence	1:1,000 in blocking buffer (1% human AB in PBS), 1 h RT

a 2°, secondary.

**TABLE 2:** Plasmids used in this study

Plasmid name	Use	Source	Bacterial selection cassette	Mammalian selection cassette(s) (lentiviral plasmids only)
pLJM-1-EV	Control vector for lentiviral expression studies	C. McCormick (Dalhousie University)	Ampicillin	Blasticidin, puromycin
pLJM-1 KapB	Lentiviral expression of KapB	C. McCormick (Dalhousie University)	Ampicillin	Blasticidin
pLKO-(shRNA)	Lentiviral expression of shRNAs (shRNA sequences in Table 3)	Cloned from pLKO-TRC (Addgene; catalog no. 26655)	Ampicillin	Puromycin
pLJM-1 α-actinin-1-GFP	Lentiviral expression of α-actinin-1–GFP	Cloned from pEGFP-N1 α-actinin-1 (Addgene; catalog no. 11908)	Ampicillin	Puromycin
pLJM-1-YAP-5SA (CA-YAP)	Lentiviral expression of constitutively active YAP	Cloned from p2XFLAG-YAP-5SA (Donated by C. McCormick; Dalhousie University)	Ampicillin	Blasticidin
pcDNA3.1	Transfection control	Invitrogen	Ampicillin	NA
pcDNA3.1 KapB	Transfection of KapB	C. McCormick (Dalhousie University)	Ampicillin	NA^*a*^
p1XFLAG	Transfection control	Cloned from p2XFLAG-YAP-5SA; donated by C. McCormick (Dalhousie University)	Ampicillin	NA
p2XFLAG-YAP 5SA	Transfection of YAP 5SA	Donated by C. McCormick (Dalhousie University)	Ampicillin	NA
pV5-YAP 6SA	Transfection of YAP 6SA	Addgene (catalog no. 42562)	Ampicillin	NA
pMD2.G	Envelope protein for lentiviral production	Addgene (catalog no. 12259)	Ampicillin	NA
psPAX2	Packaging proteins for lentiviral production	Addgene (catalog no. 12260)	Ampicillin	NA

a NA, not applicable.

**TABLE 3:** shRNA sequences used in this study

Target	Sequence (5′–3′)
Nontargeting sense	CCGGAGCACAAGCTGGAGTACAACTACTCGAGATCAACATGAGGTCGAACACGATTTG
Nontargeting antisense	AATTCAAAAAGCACAAGCTGGAGTACAACTAATCAACATGAGGTCGAACACGATTTG
mDia1 sh1 sense	CCGGCCAATTCTGCTCATAGAAATTCTCGAGAATTTCTATGAGCAGAATTGGTTTTTG
mDia1 sh1 antisense	AATTCAAAAACCAATTCTGCTCATAGAAATTCTCGAGAATTTCTATGAGCAGAATTGG
mDia1 sh2 sense	CCGGAAGATGACGTTGTTACACTTCCTCGAGGAAGTGTAACAACGTCATCTTTTTTTG
mDia1 sh2 antisense	AATTCAAAAAAAGATGACGTTGTTACACTTCCTCGAGGAAGTGTAACAACGTCATCTT
ROCK1 sh1 sense	CCGGAAGATGACGTTGTTACACTTCCTCGAGGAAGTGTAACAACGTCATCTTTTTTTG
ROCK1 sh1 antisense	AATTCAAAAAAAGATGACGTTGTTACACTTCCTCGAGGAAGTGTAACAACGTCATCTT
ROCK1 sh2 sense	CCGGAAGATGACGTTGTTACACTTCCTCGAGGAAGTGTAACAACGTCATCTTTTTTTG
ROCK1 sh2 antisense	AATTCAAAAAAAGATGACGTTGTTACACTTCCTCGAGGAAGTGTAACAACGTCATCTT
ROCK2 sh1 sense	CCGGCGTTGCCATATTAAGTGTCATCTCGAGATGACACTTAATATGGCAACGTTTTTG
ROCK2 sh1 antisense	AATTCAAAAACGTTGCCATATTAAGTGTCATCTCGAGATGACACTTAATATGGCAACG
ROCK2 sh2 sense	CCGGGCCTTGCATATTGGTCTGGATCTCGAGATCCAGACCAATATGCAAGGCTTTTTG
ROCK2 sh2 antisense	AATTCAAAAAGCCTTGCATATTGGTCTGGATCTCGAGATCCAGACCAATATGCAAGGC
Cofilin sh1 sense	CCGGACGACATGAAGGTGCGTAAGTCTCGAGACTTACGCACCTTCATGTCGTTTTTTG
Cofilin sh1 antisense	AATTCAAAAAACGACATGAAGGTGCGTAAGTCTCGAGACTTACGCACCTTCATGTCGT
Cofilin sh2 sense	CCGGCCAGATAAGGACTGCCGCTATCTCGAGATAGCGGCAGTCCTTATCTGGTTTTTG
Cofilin sh2 antisense	AATTCAAAAACCAGATAAGGACTGCCGCTATCTCGAGATAGCGGCAGTCCTTATCTGG
YAP sh1 sense	CCGGCTGGTCAGAGATACTTCTTAACTCGAGTTAAGAAGTATCTCTGACCAGTTTTTC
YAP sh1 antisense	AATTGAAAAACTGGTCAGAGATACTTCTTAACTCGAGTTAAGAAGTATCTCTGACCAG
YAP sh2 sense	CCGGAAGCTTTGAGTTCTGACATCCCTCGAGGGATGTCAGAACTCAAAGCTTTTTTTC
YAP sh2 antisense	AATTGAAAAAAAGCTTTGAGTTCTGACATCCCTCGAGGGATGTCAGAACTCAAAGCTT

**TABLE 4:** Drug treatments used in this study

Drug	Use	Source (catalog no.)	Concn(s) used	Duration
Y-27623 dihydrochloride (ROCKi)	Nonisoform specific inhibition of ROCK	Sigma-Aldrich (Y0503)	10 μM	4 h
Jasplakinolide	Aberrant polymerization of actin, decreasing monomeric G-actin	Sigma-Aldrich (J4580)	0.5 μM, 1 μM	30 min
Cytochalasin D	Inhibition of actin polymerization, increasing monomeric G-actin	Sigma-Aldrich (C8273)	1 μg/ml	30 min
(–)-Blebbistatin	Inhibition of MLC contractility	Sigma-Aldrich (B0560)	10 μM	30 min
Calyculin A	Inhibition of MLC phosphatase, resulting in cell contraction	Abcam (ab141784)	2.5 nM, 5 nM	20 min
Bafilomycin A1	Inhibition of autophagosome-lysosome fusion, blocking final degradative step of autophagy	Sigma-Aldrich (B1793)	10 nM	30 min

### Cell cultures.

Human embryonic kidney 293T and 293A cells (HEK-293T and HEK-293A, respectively; ATCC, Manassas, VA, USA) and human cervical adenocarcinoma cells expressing a tetracycline-inducible repressor (HeLa Tet-Off; Clontech, Mountain View, CA, USA) were cultured in Dulbecco’s modified Eagle medium (DMEM; Gibco, Carlsbad, CA, USA) supplemented with 10% heat-inactivated fetal bovine serum (Gibco), 100 U/ml penicillin, 100 μg/ml streptomycin, and 2 mM l-glutamine (Gibco). Pooled human umbilical vein endothelial cells (HUVECs; Lonza, Basel, Switzerland) were cultured in endothelial cell growth medium 2 (EGM-2; Lonza). For HUVEC passaging, tissue culture plates were precoated for 30 min at 37°C with 0.1% (wt/vol) porcine gelatin (Sigma, St. Louis, Missouri, USA) in 1× phosphate-buffered saline (PBS; Gibco). All cells were routinely tested for mycoplasma by PCR method.

### Transfection for lentivirus production.

HEK-293T cells at 70 to 80% confluence were transfected using 3.3 μg of the target lentiviral construct, 2 μg pSPAX2, and 1 μg pMD2.G with 1 mg/ml polyethylenimine (PEI; Sigma) in serum-free DMEM. After 5 to 6 h, medium was replaced with antibiotic-free DMEM containing 10% fetal bovine serum (FBS) and 2 mM l-glutamine (Gibco). Transfected cells were incubated for 48 h at 37°C to allow lentivirus production. The supernatant media containing viral particles was harvested and filtered through a 0.45-μm polyethersulfone (PES) filter (VWR, Radnor, PA, USA) and aliquoted. Virus was stored at −80°C until use.

### Lentiviral transduction.

Lentivirus was supplied into wells of plated HUVECs in EGM-2 supplemented with 5 μg/ml hexadimethrine bromide (Polybrene). After 24 h of incubation, cells were selected with either 5 μg/ml blasticidin (Sigma) for 96 h, replacing the media and antibiotic at 48 h, or 1 μg/ml puromycin (Sigma) for 48 h. Following selection, HUVEC medium was replaced with EGM-2 without selection for at least 24 h recovery before further use. Lentivirus was titrated to ensure no significant changes in cell viability from nontargeting control.

### Immunofluorescence.

Immunofluorescence was performed as described previously (45). Briefly, cells were grown on coverslips (no. 1.5; Electron Microscopy Sciences, Hatfield, PA, USA). Following treatment, coverslips were fixed in 4% paraformaldehyde (PFA; Electron Microscopy Sciences) in PBS at 37°C for 10 min, permeabilized with 0.1% Triton X-100 (Sigma) in 1× PBS for 10 min at room temperature (RT), and blocked in 1% human AB serum (blocking buffer; Sigma) in 1× PBS for 1 h at RT. An exception to this is samples being prepared for staining endogenous LC3, which were fixed and permeabilized in ice-cold methanol prior to blocking. Coverslips were then incubated with diluted primary antibody in blocking buffer overnight at 4°C in a humidified chamber. After primary antibody incubation, coverslips were washed with 1× PBS and then incubated in fluorescently tagged secondary antibody diluted in blocking buffer for 1 h at RT. If applicable, coverslips were stained with phalloidin-conjugated Alexa Fluor 647 (Invitrogen; 1:100) in 1× PBS for 1.5 h. Coverslips were mounted onto microscope slides (Fisherbrand, Pittsburgh, PA, USA) using Prolong Gold antifade mounting media (Invitrogen, Carlsbad, CA, USA). For coverslips that were used for EDC4 puncta quantification, the following modifications to immunofluorescence were made: (i) prior to permeabilization, coverslips were stained with wheat germ agglutinin (WGA)-Alexa Fluor 647 conjugate (Invitrogen; 1:400) in 1× PBS for 10 min at RT, and (ii) following secondary antibody incubation, coverslips were stained with 4′,6-diamidino-2-phenylindole (DAPI; Invitrogen; 1:10,000) in 1× PBS for 5 min.

Confocal imaging was performed on the Zeiss LSM 880 confocal microscope (Charbonneau Microscopy Facility, University of Calgary, Calgary, Canada) at the 63× oil objective. CellProfiler imaging was performed on the Zeiss AxioImager Z2 (CORES facility, Dalhousie University, Halifax, Canada) or Zeiss AxioObserver (Charbonneau Microscopy Facility, University of Calgary) at the 40× oil objective.

### Quantification of processing bodies using CellProfiler analysis.

CellProfiler (https://cellprofiler.org) is an open-source software for high-content image analysis (142) and was used to develop an unbiased method for quantifying changes to PB dynamics. The pipeline used for quantifying PBs was structured as follows. To detect nuclei, the DAPI image was thresholded into a binary image. In the binary image, primary objects between 30 to 200 pixels in diameter were detected and defined as nuclei. Cells were identified as secondary objects in the WGA image using a propagation function from the identified nuclei, which determined the cell’s outer edge. Using the parameters of a defined nucleus and cell border, the cytoplasm was then defined as a tertiary object. The EDC4 channel image was enhanced using an “enhance speckles” function to identify distinct puncta and eliminate background staining. The cytoplasm image was then applied as a mask to the enhanced puncta image to ensure quantitation of only cytoplasmic puncta. EDC4 puncta were measured in the cytoplasm of cells using a “global thresholding with robust background adjustments” function as defined by the program. The threshold cutoff for identified EDC4 puncta remained constant between all experiments with identical staining parameters. Puncta number per cell, intensity, and locations with respect to the nucleus were measured and exported as csv files and analyzed in RStudio. Data were represented as fold change in EDC4 puncta count per cell normalized to the vector puncta count. “Relative EDC4 puncta/cell (KapB/vector)” demonstrates the KapB puncta count divided by vector puncta count, a ratio that was calculated within each treatment group for each biological replicate.

### Protein electrophoresis and immunoblotting.

Cells were lysed in 2× Laemmli buffer (20% glycerol, 4% SDS, and 120 mM Tris-HCl), between 150 to 300 μl, depending on cell density. Lysates were homogenized with a 0.21-gauge needle and supplemented to contain 0.02% (wt/vol) bromophenol blue (Sigma) and 0.05 M dithiothreitol (DTT; Sigma) and then heated at 95°C for 5 min. We cast 7.5 or 12% TGX stain-free SDS-polyacrylamide gels (Bio-Rad) according to the instructions of the manufacturer, and 5 to 15 μg of total protein were subjected to SDS gel electrophoresis using 1× SDS running buffer (25 mM Tris, 192 mM glycine, and 0.1% SDS). Precision Plus Protein all blue prestained protein standards (Bio-Rad, Hercules, CA, USA) was used as a molecular weight marker. After electrophoresis, gels were UV activated using the ChemiDocTouch (Bio-Rad) stain-free gel setting with automated exposure for 45 s. The protein was transferred to low-fluorescence polyvinylidene difluoride (PVDF) membranes (Bio-Rad) on the Trans-Blot Turbo transfer system (Bio-Rad) according to the instructions of the manufacturer. Following transfer, total protein amounts on membranes were imaged on the ChemiDocTouch using the stain-free membrane setting with automated exposure. Membranes were then blocked using 5% bovine serum albumin (BSA) (Sigma) in 1× TBST (150 nM NaCl, 10 mM Tris [pH 7.8], and 0.01% Tween 20) for 1 h at RT. Primary antibody was diluted in 2.5% BSA in 1× TBST. Membranes were incubated in primary antibody solution overnight at 4°C with rocking. The following day, membranes were washed 3 times for 5 min in 1× TBST. Membranes were incubated with the appropriate secondary antibody and conjugated to horseradish peroxidase (HRP) for 1 h at RT. Membranes were washed 4 times for 5 min in 1× TBST. Clarity Western ECL blotting substrate (Bio-Rad) was mixed at a 1:1 ratio and applied to the membrane for 5 min. Chemiluminescent signal was imaged on ChemiDocTouch chemiluminescence setting. Band intensity was quantified using Image Lab software (Bio-Rad), normalizing to total protein.

### Filamentous and globular actin fractionation.

Actin stabilization and depolymerization buffer recipes were obtained from Rasmussen et al. (84), and protocol was adapted from G-actin/F-actin in vivo assay kit (143). Briefly, following treatment, HUVECs were lysed in 100 μl actin stabilization buffer {0.1 M PIPES [piperazine-*N*,*N*′-bis(2-ethanesulfonic acid)] (pH 6.9), 30% glycerol, 5% dimethyl sulfoxide (DMSO), 1 mM MgSO_4_, 1 mM EGTA, 1% Triton X-100, 1 mM ATP, and protease inhibitor} for each well of a confluent 6-well dish. Lysates were homogenized with a 21-gauge needle, incubated at 30°C for 10 min, and centrifuged at 350 × *g* for 5 min to remove cell debris. Supernatant was then transferred to a 600-μl thick-walled centrifuge tube (Beckman, CA, USA) and subjected to 100,000 × *g* ultracentrifugation for 1 h. Following ultracentrifugation, the supernatant (G-actin) was mixed with 25 μl 5× SDS sample buffer (0.25 M Tris-Cl [pH 6.8], 10% SDS, 50% glycerol, 0.5 M DTT, and 0.25% bromophenol blue) and incubated at 95°C for 5 min. The pellet from ultracentrifugation (F-actin) was mixed with ice-cold 100-μl actin depolymerization buffer (0.1 M PIPES [pH 6.8], 1 mM MgSO_4_, 10 mM CaCl2, and 5 μM cytochalasin D). The F-actin fraction was then mixed with 25 μl 5× SDS sample buffer and incubated at 95°C for 5 min. Samples were subjected to protein electrophoresis and immunoblotting using antibodies for β-actin.

### Quantitative reverse transcriptase PCR.

Cells were lysed in 250 μl RLT buffer (Qiagen, Hilden, Germany), and RNA was extracted using the RNeasy Plus minikit (Qiagen) according to the manufacturer’s instructions. cDNA was synthesized from 1 μg of total RNA using the qScript cDNA supermix (QuantaBio, Hilden, Germany) according to the manufacturer’s instructions. Real-time quantitative PCR with SsoFast EvaGreen qPCR mastermix (Bio-Rad) was used to quantify the fold change in mRNA abundance. Relative fluorescence was quantified using CFX Connect (Bio-Rad). All quantitative reverse transcriptase PCR (qRT-PCR) primer efficiencies were between 90 and 110% in HUVECs, and sequences are found in Table 5.

**TABLE 5:** qRT-PCR primers used in this study

Target(s)	Forward or reverse	Sequence	*T_m_* (°C)^*a*^	Reference
CYR61	Forward	ATGGTCCCAGTGCTCAAAGA	60	19
CYR61	Reverse	GGGCCGGTATTTCTTCACAC	62	19
CTGF	Forward	CAGCATGGACGTTCGTCTG	60	19
CTGF	Reverse	AACCACGGTTTGGTCCTTGG	62	19
CTGF	Forward	CCCTCGCGGCTTACCG	56	19
CTGF	Reverse	GGACCAGGCAGTTGGCTCT	62	19
ANKRD1	Forward	ACGCCAAAGACAGAGAAGGA	60	19
ANKRD1	Reverse	TTCTGCCAGTGTAGCACCAG	52	19
ARMUS/TBC1D2	Forward	GTGTCTCCCTTTGGGAAGCTG	61	
ARMUS/TBC1D2	Reverse	TGGATCCCTGGCAGACTCTT	60	
HPRT-1	Forward	CTTTCCTTGGTCAGGCAGTATAA	66	131
HPRT-1	Reverse	AGTCTGGCTTATATCCAACACTTC	60	131
HPRT-1	Forward	TGGCGTCGTGATTAGTGATG	64	131
HPRT-1	Reverse	GACGTTCAGTCCTGTCCATAAT	68	131

a *T_m_*, melting temperature.

### Luciferase assay for TEAD transcriptional activity.

HEK-293A cells were seeded in antibiotic-free DMEM at 75,000 cells/well. Mixtures of 500 ng of the target construct (pcDNA [vector], pcDNA-KapB [KapB], p1XFLAG, or p2XFLAG-YAP 5SA), 450 ng 8×GTIIC luciferase reporter, 50 ng (transcription-export) (TREX)-*Renilla* luciferase reporter, and 3 μl FuGENE HD transfection reagent (Promega, Madison, WI, USA) were transfected into HEK-293A cells. After 36 h, DMEM containing only 2 mM l-glutamine (starvation media) was supplied to the cells. Twelve hours after addition of starvation media, cells were lysed in 200 μl passive lysis buffer (Promega), and luciferase activity was assayed using the dual-luciferase reporter assay system (Promega) according to the manufacturer’s instructions. Luminescence was measured using the GloMax luminometer (Promega).

### Luciferase assay for stability of mRNA with AU-rich elements.

This technique is described in Corcoran et al. (102). Briefly, HeLa Tet-Off cells were seeded in antibiotic-free DMEM at 100,000 cells/well. Mixtures of 900 ng of the target construct (pcDNA [vector], pcDNA-KapB [KapB], p1XFLAG, or p2XFLAG-YAP 5SA), 90 ng TREX-Firefly ARE luciferase, 10 ng TREX-*Renilla* luciferase, and 3 μl FuGENE HD transfection reagent (Promega) were transfected into HeLa Tet-Off cells. After 36 h, 1 μg/ml doxycycline was supplied to the cells to inhibit further transcription of each reporter. Twelve hours after addition of doxycycline, cells were lysed in 200 μl passive lysis buffer (Promega), and luciferase activity was assayed using the dual-luciferase reporter assay system (Promega) according to the manufacturer’s instructions. Luminescence was measured using the GloMax luminometer (Promega).

### Collagen coating for altering matrix stiffness.

Coverslips (no. 1.5, Electron Microscopy Sciences) were coated with a dilution series (0 to 64 μg/cm^2^) of rat tail collagen 1 (Gibco) in 0.02 M acetic acid for 4 h at RT. Slides were sterilized with UV irradiation and washed 2 times with sterile 1× PBS prior to seeding cells.

### Unidirectional fluid flow for endothelial cell shear stress.

A parallel-plate flow chamber was used to expose ECs to shear stress. The system was described in detail in Gomez-Garcia et al. Briefly, cleaned, unfrosted microscope slides (Cole-Parmer, Vernon Hills, IL, USA) were coated for 4 h at RT with rat tail collagen 1 (Gibco) in 0.02 M acetic acid for a resultant 8.3 μg/cm^2^ collagen density. Slides were sterilized with UV irradiation and washed 2 times with sterile 1× PBS. HUVECs were seeded at a density of 350,000 cells/slide and cultured for 24 h. Forty-five milliliters of EGM-2 supplemented with dextran (Spectrum Chemical, New Brunswick, NJ, USA) for a resultant 3-cP viscosity was added to the stock medium bottle. The stock medium bottle was connected with the associated tubing and pulse dampener. Slides with seeded cells were inserted onto the flow chamber, a gasket (Specialty Manufacturing, Calgary, Canada) was added, and the system was sealed shut and attached to the flow loop following the outlet of a pulse dampener. The rate of fluid flow was started at 0.3 liter/min and doubled every 15 min until final flow rates of 0.6 liter/min and 2.7 liter/min were reached, corresponding to shear stress rates of 2 and 10 dyn/cm^2^. Following 21 h, cells were removed and immediately fixed for immunofluorescence or lysed for immunoblotting.

### Statistical analysis.

Statistical analysis was performed in GraphPad Prism 8.0 software. Significance was determined using a ratio-paired *t* test or repeated-measures analysis of variance (ANOVA). One-tailed ratio-paired *t* tests were applied in comparisons specifically examining PB restoration in KapB-expressing cells as a directional hypothesis. In all other comparisons, two-tailed ratio-paired *t* tests were applied. Significance was determined at a *P* value of 0.05. Each biological replicate for CellProfiler quantification consisted of 6 images of each treatment in a given experiment, counting approximately 100 to 200 cells per treatment.
